# Vitamin C boosts DNA demethylation in *TET2* germline mutation carriers

**DOI:** 10.1186/s13148-022-01404-6

**Published:** 2023-01-14

**Authors:** Aurora Taira, Kimmo Palin, Anna Kuosmanen, Niko Välimäki, Outi Kuittinen, Outi Kuismin, Eevi Kaasinen, Kristiina Rajamäki, Lauri A. Aaltonen

**Affiliations:** 1 Department of Medical and Clinical Genetics, University of Helsinki, Helsinki, Finland; 2 Applied Tumor Genomics Research Program, Research Programs Unit, University of Helsinki, Helsinki, Finland; 3 iCAN Digital Precision Cancer Medicine Flagship, University of Helsinki, Helsinki, Finland; 4 Department of Clinical Medicine, Faculty of Health Sciences, University of Eastern Finland, Kuopio, Finland; 5 Cancer Center, Kuopio University Hospital, Kuopio, Finland; 6 PEDEGO Research Unit, University of Oulu, Oulu, Finland; 7 Medical Research Center Oulu, University of Oulu and Oulu University Hospital, Oulu, Finland; 8 Department of Clinical Genetics, Oulu University Hospital, Oulu, Finland

**Keywords:** DNA methylation, TET2 mutations, Vitamin C, Hematological neoplasia

## Abstract

**Background:**

Accurate regulation of DNA methylation is necessary for normal cells to differentiate, develop and function. TET2 catalyzes stepwise DNA demethylation in hematopoietic cells. Mutations in the *TET2* gene predispose to hematological malignancies by causing DNA methylation overload and aberrant epigenomic landscape. Studies on mice and cell lines show that the function of TET2 is boosted by vitamin C. Thus, by strengthening the demethylation activity of TET2, vitamin C could play a role in the prevention of hematological malignancies in individuals with TET2 dysfunction. We recently identified a family with lymphoma predisposition where a heterozygous truncating germline mutation in *TET2* segregated with nodular lymphocyte-predominant Hodgkin lymphoma. The mutation carriers displayed a hypermethylation pattern that was absent in the family members without the mutation.

**Methods:**

In a clinical trial of 1 year, we investigated the effects of oral 1 g/day vitamin C supplementation on DNA methylation by analyzing genome-wide DNA methylation and gene expression patterns from the family members.

**Results:**

We show that vitamin C reinforces the DNA demethylation cascade, reduces the proportion of hypermethylated loci and diminishes gene expression differences between *TET2* mutation carriers and control individuals.

**Conclusions:**

These results suggest that vitamin C supplementation increases DNA methylation turnover and provide a basis for further work to examine the potential benefits of vitamin C supplementation in individuals with germline and somatic *TET2* mutations.

*Trial registration*: This trial was registered at EudraCT with reference number of 2018-000155-41 (01.04.2019).

**Graphical Abstract:**

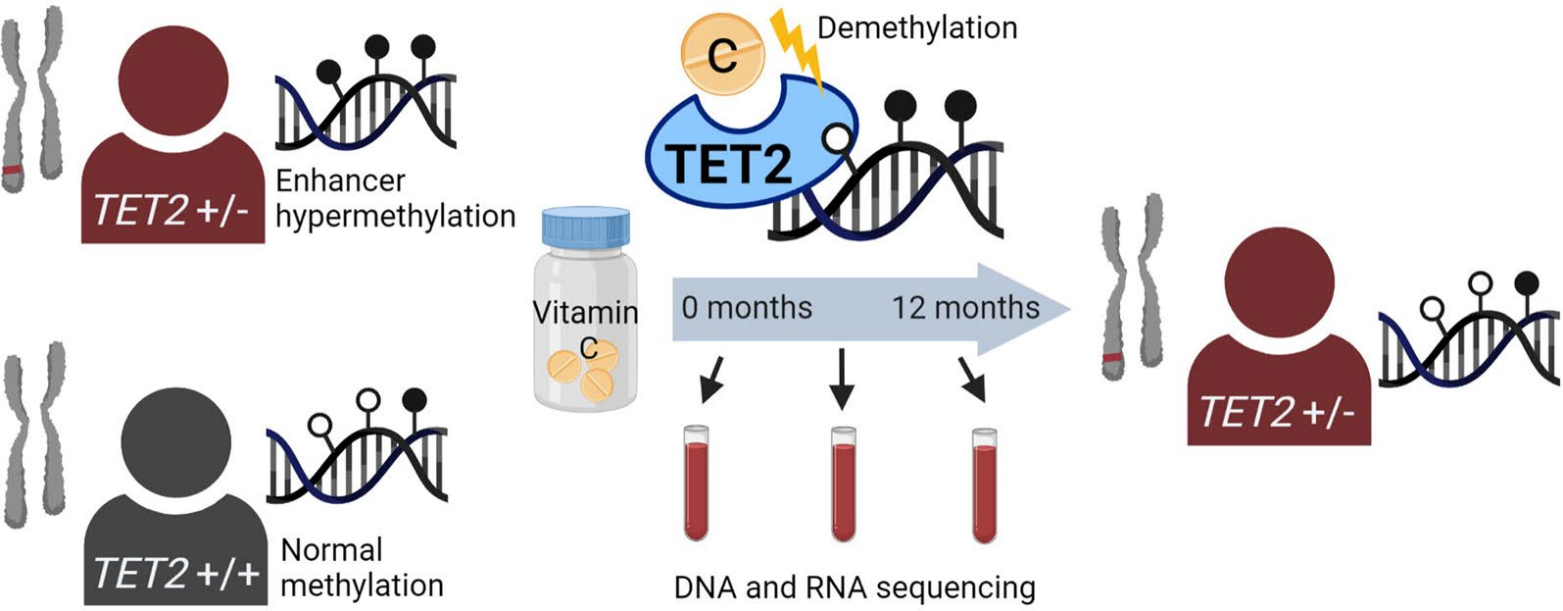

**Supplementary Information:**

The online version contains supplementary material available at 10.1186/s13148-022-01404-6.

## Background

Aberrant DNA methylation disturbs transcriptional regulation, predisposes cells to malignant transformation and is a hallmark of human cancers [1]. Inactivating somatic mutations in *TET2*, a gene regulating DNA demethylation, are common in both myeloid and lymphoid malignancies including chronic myelomonocytic leukemia, acute myeloid leukemia (AML), myelodysplastic syndrome, angioimmunoblastic T cell lymphoma and, at a lower frequency, diffuse large B cell lymphoma and mantle cell lymphoma [2]. While *TET2* loss serves as a risk factor for further malignant degeneration, *TET2* mutations alone are insufficient to trigger neoplasia [3, 4].

The human ten–eleven translocation (TET) protein family comprises three members (TET1, TET2 and TET3) that share the same catalytic activity. TETs mediate the iterative oxidation of 5-methylcytosine (5-mC) to 5-hydroxymethylcytosine (5-hmC), 5-formylcytosine (5-fC) and 5-carboxylcytosine (5-caC), initiating the demethylation of cytosine residues in DNA. Eventually, the methyl group can be removed and an unmodified cytosine restored. The structure and expression patterns of TET proteins are divergent. TET2, unlike TET1 and TET3, lacks the CpG-interacting CXXC zinc finger domain and is highly expressed in the hematopoietic system [2].

Vitamin C has been shown to boost TET-mediated 5-mC oxidation steps in cultured cells and in vivo in mice [5–7]. Mice, like many other mammals, synthesize ascorbic acid in the liver using the enzyme gulonolactone oxidase (*Gulo*), whereas humans are dependent on dietary intake of vitamin C. *Gulo* knockdown mice on an ascorbate-poor diet had significantly higher levels of hematopoietic stem cells than did *Gulo* wild-type mice, accompanied by reduced TET2 activity. Moreover, ascorbate depletion was linked to accelerated leukemogenesis. Dietary intake of ascorbate seemed to suppress these effects [8].

Vitamin C has been extensively studied as a potential cancer-preventive agent in the context of solid tumors, but the results have remained inconclusive [9]. Recent studies [5, 6, 8, 10, 11] linking vitamin C, TET2 and hematological malignancies suggest that sufficient intake of vitamin C could play a role in the prevention of these diseases. Clinical trials to examine this possibility are warranted and necessary, not least due to the differences in vitamin C metabolism between mice and men.

We have previously reported a family in which a heterozygous truncating germline mutation in *TET2* segregated with nodular lymphocyte-predominant Hodgkin lymphoma (NLPHL). Compared to unrelated control individuals, the mutation carriers displayed an excess of hypermethylation that was absent in family members without the mutation [12]. Thus, mutations in *TET2* predispose these individuals to familial DNA demethylation deficiency and hematological neoplasia. Mutation carriers express both *TET2* alleles at the mRNA level, and the expected loss of the mutated allele is seen at the protein level [12]; thus, mutation carriers harbor only one functional copy of the enzyme gene.

The family provided us with a unique opportunity to examine the effects of dietary vitamin C supplementation in individuals with TET2 deficiency. Three unaffected *TET2* mutation carriers, two elderly mutation carriers previously affected by lymphoma, and three control individuals from the family participated in this non-randomized, open-label clinical trial of vitamin C on the DNA methylation load. Participants took 1 g of vitamin C as a dietary supplement daily for 1 year. Through analysis of genome-wide methylation and gene expression patterns, we found promising effects of long-term vitamin C supplementation in unaffected TET2-deficient individuals.

## Methods

### Study design

**Fig. 1:**
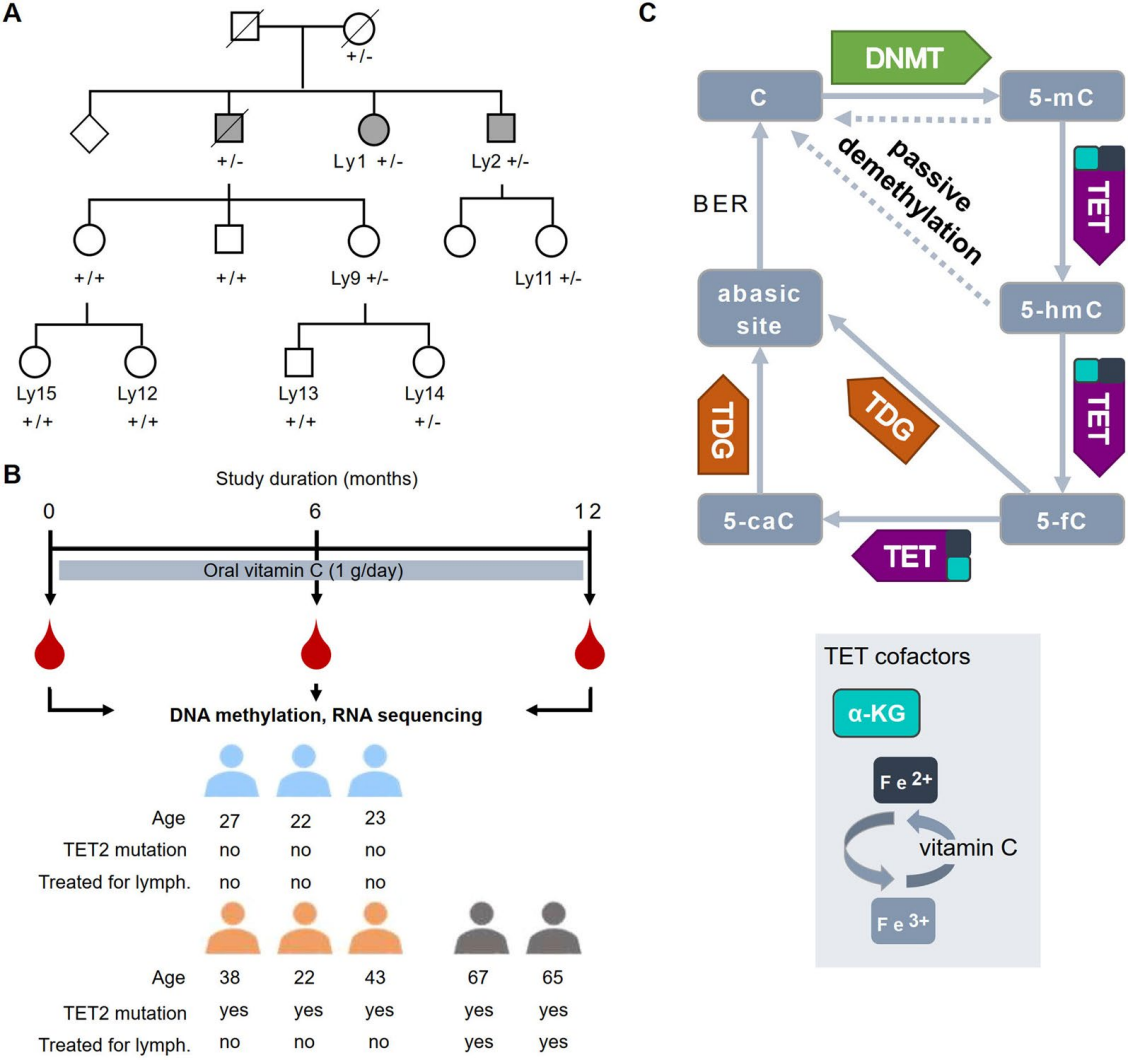
Study of vitamin C supplementation in individuals with germline *TET2* mutations. **A** Pedigree (details modified for confidentiality) showing *TET2* c.4500delA mutation statuses (+ / + control, +/− mutation carrier) and the individuals previously treated for nodular lymphocyte-predominant Hodgkin lymphoma (filled symbols). Males (square); females (circle). The individuals sampled for this study are marked with a 'Ly' patient ID. **B** A schematic drawing showing the study plan and information on the study individuals. **C** Regulation of DNA methylation. Cytosine (**C**) is methylated by DNA methyltransferases (DNMTs) into 5-methylcytosine (5-mC). Demethylation can occur passively (dashed lines) during cell division or actively via oxidation by the ten–eleven translocation (TET) enzymes, including TET2, resulting in generation of 5-hydroxymethylcytosine (5-hmC) and its further oxidation products 5-formylcytosine (5-fC) and 5-carboxylcytosine (5-caC). TETs are dependent on the availability of cofactors α-ketoglutarate (α-KG) and Fe^2+^. Ascorbic acid (vitamin C) enhances TET activity, likely by maintaining the redox state of Fe^2+^, resulting in increased production of all 5-mC oxidation products [5–7, 10]. Only 5-fC and 5-caC can be actively converted to unmethylated C via excision by thymine DNA glycosylase (TDG). The resulting abasic site is repaired into C via the base excision repair (BER) pathway

The study subjects were recruited from a previously characterized family [12] segregating a heterozygous *TET2* NM_001127208.2:c.4500delA loss-of-function mutation (Fig. 1A). Three mutation carriers and three mutation-negative controls from the younger generations formed the core of this study. Two additional mutation carriers (Ly1 and Ly2) were elderly and had been diagnosed with and treated for NLPHL. No signs of active disease have emerged for several years. The study subjects were interviewed regarding their use of vitamin C supplementation prior to the trial. One unaffected mutation carrier had undergone a gastric bypass surgery a year before the trial and thus was taking 580 mg of vitamin C daily. One individual previously treated for lymphoma and one control individual reported they had been taking multivitamin pills containing 80 mg of vitamin C daily.

During the 1-year trial, 1 g of vitamin C was administered daily as a single oral dose. Complete plasma saturation has been reported to occur at 1 g daily [13]. Blood samples were collected from all study participants at baseline before vitamin C supplementation (0 months), and after 6 months and 12 months of daily vitamin C administration (Fig. 1B). Nanopore long-read sequencing for calling genome-wide DNA methylation and hydroxymethylation was combined with RNA-sequencing of the whole blood samples at each time point. The role of TET enzymes (TET1, TET2 and TET3) in DNA demethylation and the effect of vitamin C on TET enzyme activity are summarized in Fig. 1C. In this trial, we hypothesized that vitamin C supplementation might boost the enzymatic activity of the functional wild-type TET2 at the protein level, to reverse the negative effects of TET2 haploinsufficiency. By interacting with other members of the demethylation cascade, vitamin C could also indirectly affect the activity of TET enzymes. The study has been registered with the EudraCT reference number 2018-000155-41. The samples and patient information were obtained with approval from the ethics committee of the Hospital District of Helsinki and Uusimaa, the Northern Ostrobothnia Hospital District as well as National Supervisory Authority for Welfare and Health. Signed informed consent was obtained from all study participants. All authors had access to primary clinical trial data.

### Nanopore sequencing

DNA samples isolated from whole blood were sequenced with the Genomic DNA by Ligation (SQK-LSK109) protocol with FLO-PRO002 flowcell as per the manufacturer’s instructions (Oxford Nanopore Technologies). Sequencing and base calling were performed on the PromethION platform using MinKnow-20.06.9 including Guppy 4.0.11 high-accuracy basecalling. Subsequently, reads were aligned with minimap2 (v.2.16; preset: map-ont) [14] against the GRCh38 reference genome (GCA_000001405.15, excluding alt contigs). Data quality was inspected with NanoStat (v.1.1.2) and NanoPlot (v.1.20.0) [15]

### Methylation calling

**Fig. 2:**
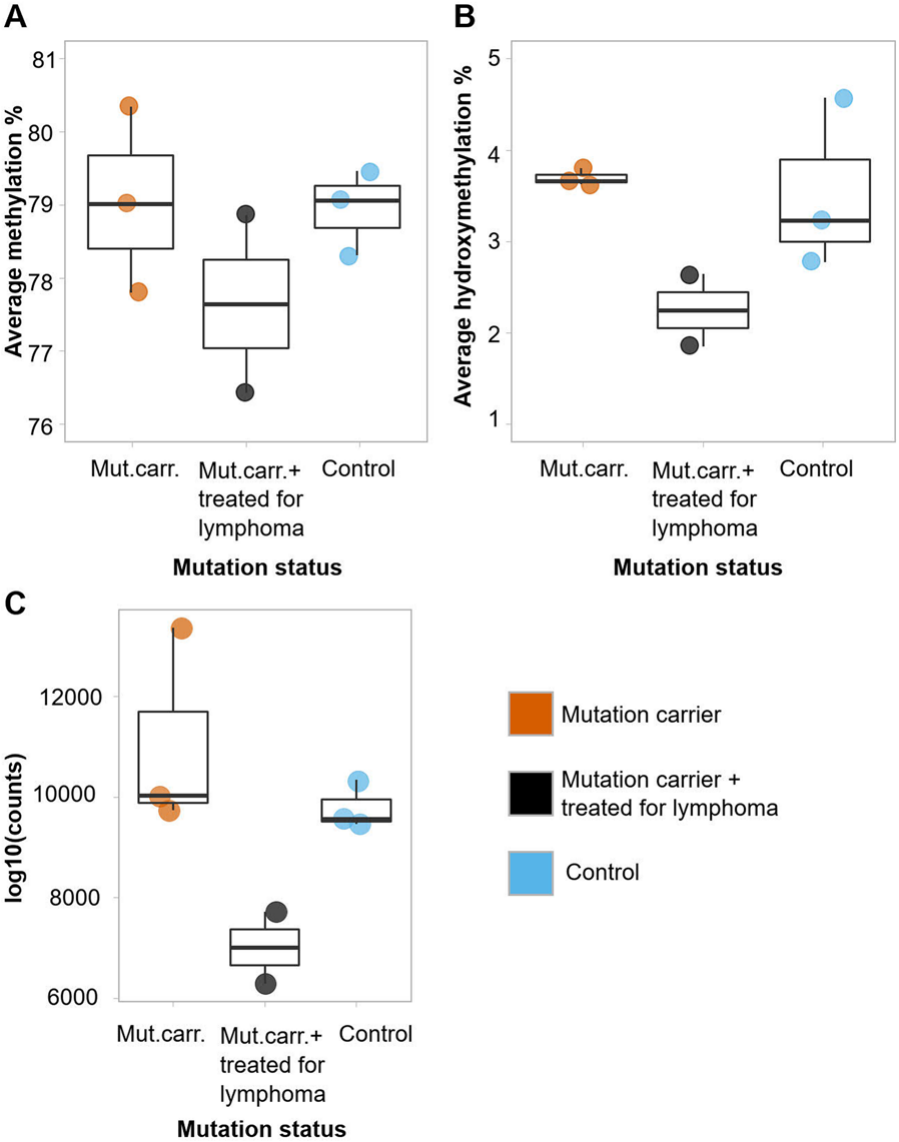
Genome-wide methylation, hydroxymethylation and expression levels of *TET2* in the study individuals at baseline. **A** Average methylation and **B** hydroxymethylation of DNA at baseline before vitamin C supplementation (0 months) in *TET2* mutation carriers versus control family members. **C** *TET2* expression from RNA-sequencing at baseline before vitamin C supplementation (0 months)

**Fig. 3:**
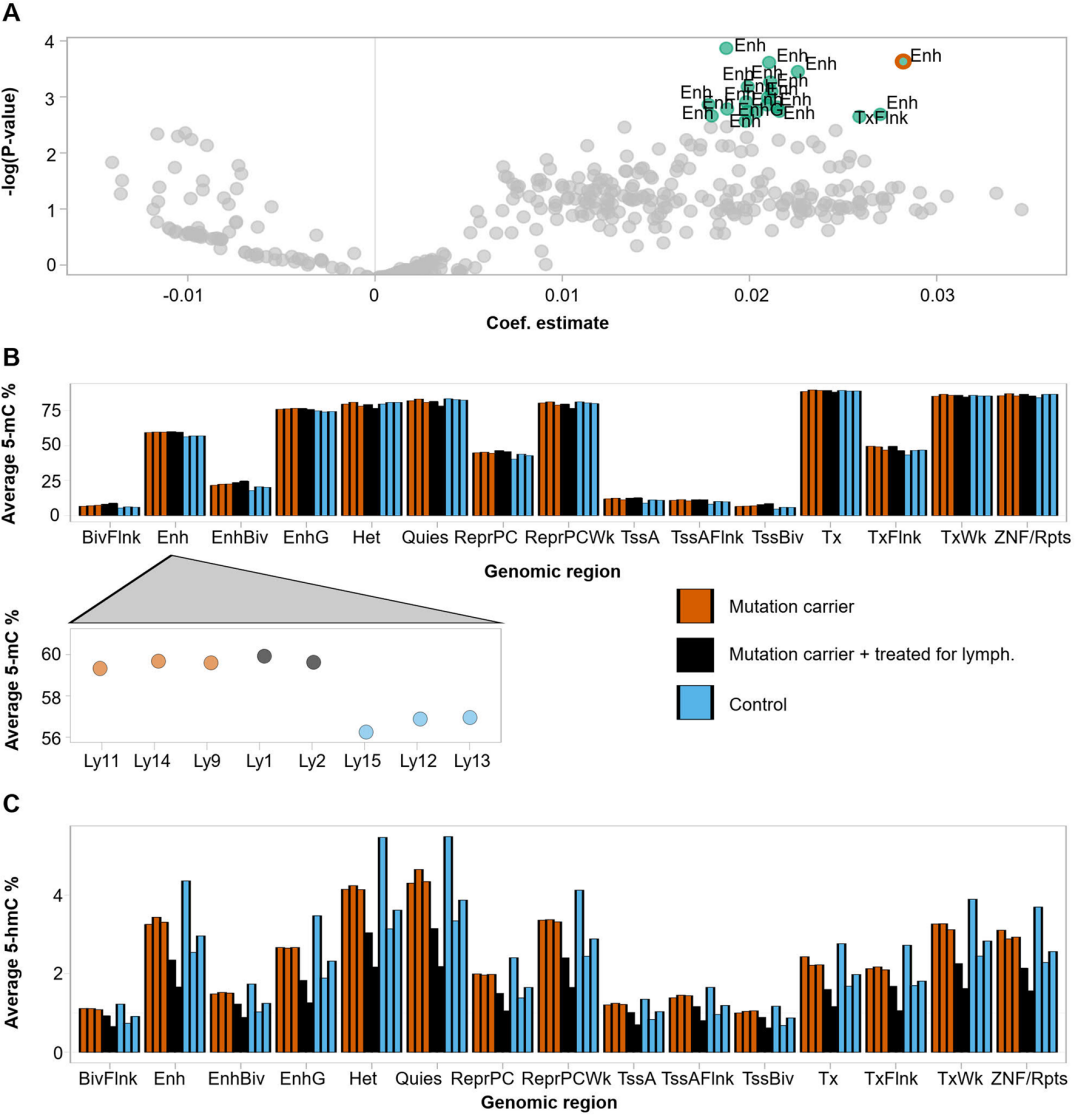
Effect of *TET2* germline mutation on methylation and hydroxymethylation at different genomic regions. **A** Volcano plot showing the effect of *TET2* mutation status on average methylation in different cell types (Additional file 1: Table S1 and Additional file 3: Table S3) and genomic annotations. Model lm(average methylation ~ mutation status + lymphoma status + genome-wide average methylation) was fitted over each cell type and annotation separately. Each point represents results related to mutation status from one cell type and annotation. Colored and labeled points showed FDR-corrected *p* value < 0.05. The result from PBMC enhancers is depicted with a red outline. Mutation status seems to increase the observed methylation average especially on enhancer (Enh) regions in multiple cell types. **B** Average DNA methylation levels before the vitamin C trial across different genomic annotations originating from peripheral blood mononuclear primary cells. Mutation carriers showed high methylation levels compared to those of the control individuals especially on enhancer regions. **C** Average hydroxymethylation levels across the annotations. Note the different *Y*-scales in panels (**B**) and (**C**). Abbreviations: *BivFlnk* Flanking bivalent TSS/enhancer, *Enh* Enhancers, *EnhBiv* Bivalent enhancer, *EnhG* Genic enhancer, *Het* Heterochromatin, *Quies* Quiescent/Low, *ReprPC* Repressed PolyComb, *ReprPCWk* Weak repressed PolyComb, *TssA* Active TSS, *TssAFlnk* Flanking active TSS, *TssBiv* Bivalent/Poised TSS, *Tx* Strong transcription, *TxFlnk* Transcribed at gene 5′ and 3′, *TxWk* Weak transcription, *ZNF/Rpts* ZNF genes and repeats

**Fig. 4:**
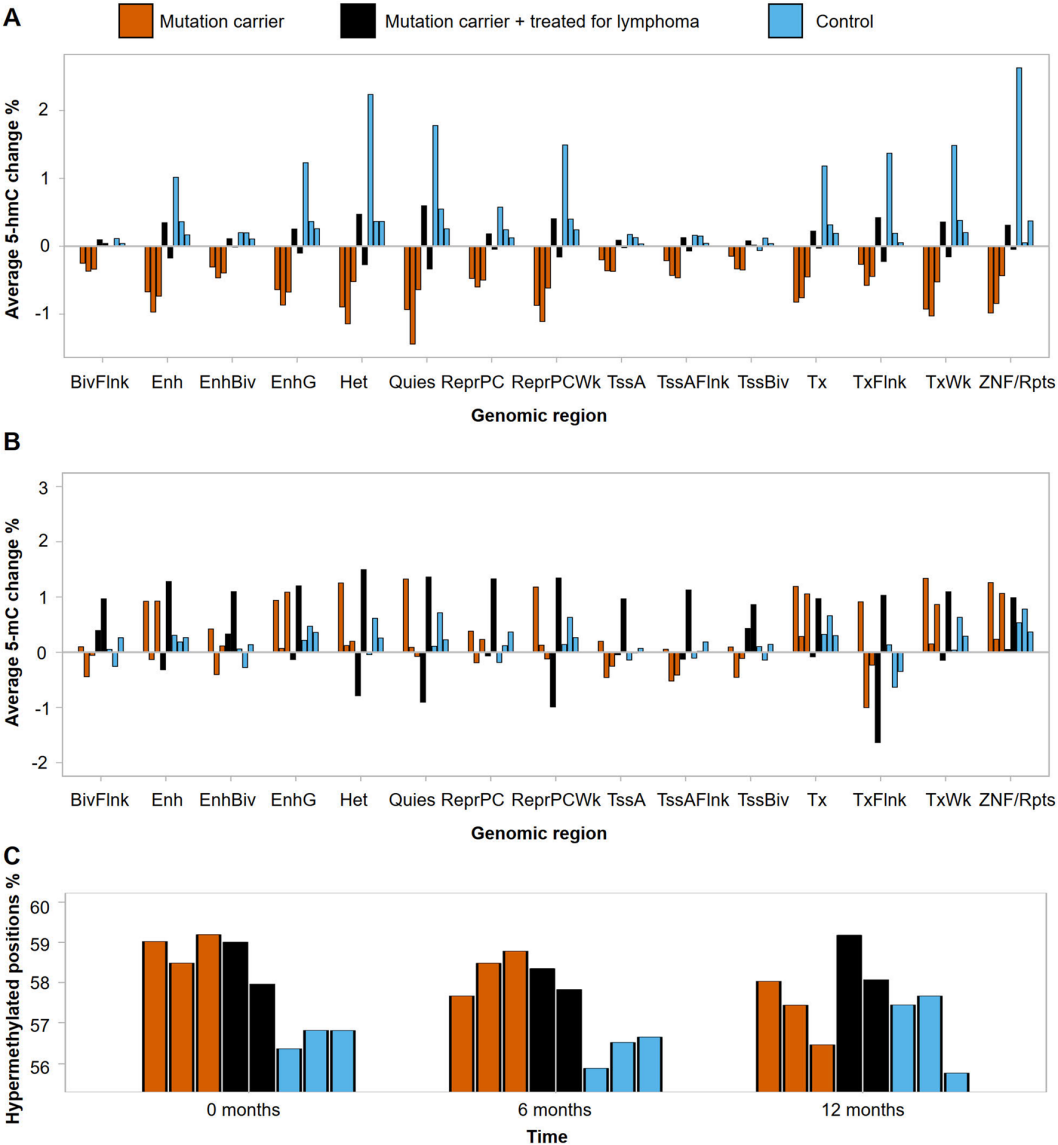
Effect of vitamin C supplementation on DNA methylation and hydroxymethylation. **A** Average change in DNA hydroxymethylation after 12 months of vitamin C supplementation at PBMC annotations. **B** Average change in DNA methylation after 12 months of vitamin C supplementation at PBMC annotations. **C** Percentage of hypermethylated positions at PBMC enhancers at different time points

**Fig. 5:**
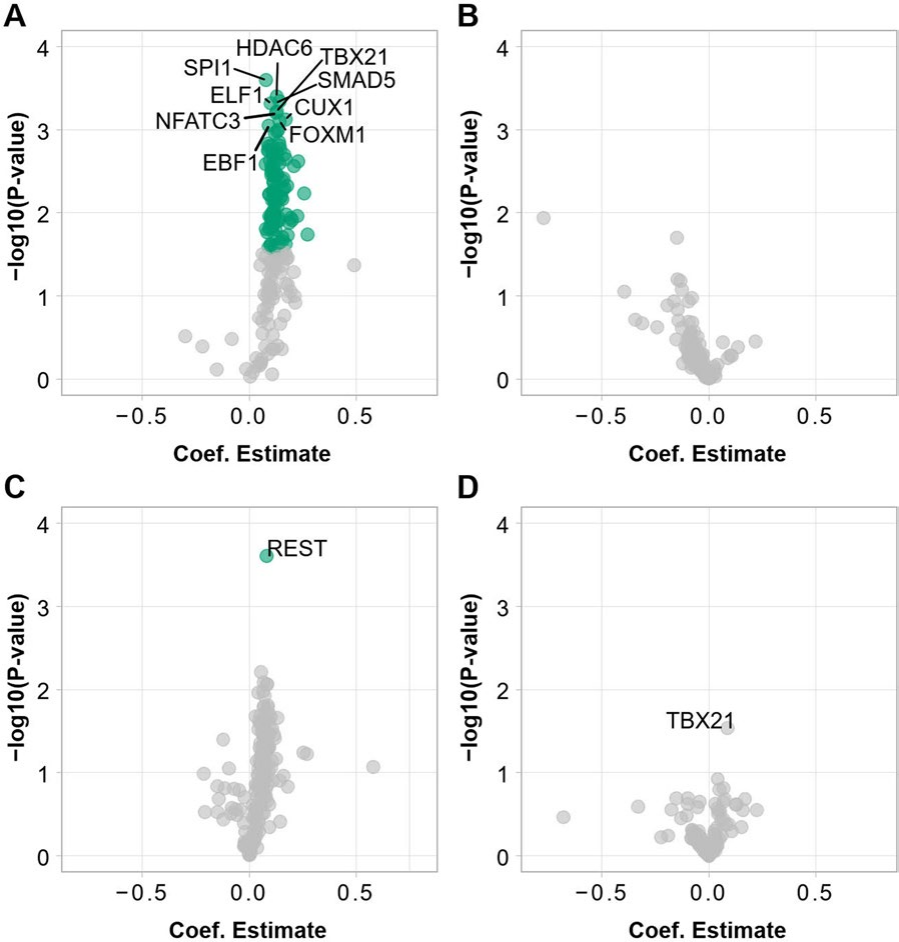
Effect of *TET2* germline mutation on methylation and hydroxymethylation at transcription factor binding sites. **A** Volcano plots showing the effect of *TET2* mutation status on average methylation at binding sites of different transcription factors overlapping PBMC enhancers at 0 months and **B** at 12 months. Average binding site methylation was defined using hypermethylated positions. Colored points had an FDR-corrected *p* value < 0.05. The most significant results are labeled with the transcription factor name. **C** Volcano plots showing the effect of *TET2* mutation status on average hydroxymethylation at binding sites of different transcription factors at 0 months and **D** at 12 months

**Fig. 6:**
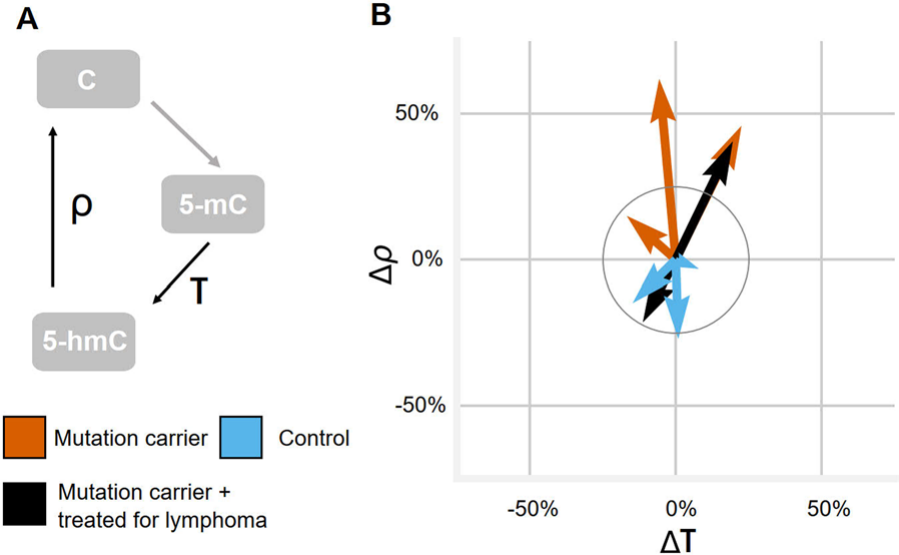
Dynamics of DNA methylation over time with vitamin C supplementation. **A** Schematic of the methylation/demethylation cycle underlying the reaction rate model. **B** Two-dimensional arrow plot of the change of rates for the reaction rate model on PBMC enhancer regions. Each arrow stands for an individual, colored by mutation status. The head of the arrow is at percent change of *τ* (first oxidation step rate) and *ρ* (further oxidation and repair rate) from 0- to 12-month time point

5-mC modification for each reference genome CpG site was called with nanopolish 0.12.5 with LLR threshold 2.5 [16]. The 5-hmC (and 5-mC) modifications were called with megalodon 2.2.9 (https://github.com/nanoporetech/megalodon) using Guppy 4.2.2 and rerio configuration res_dna_r941_min_modbases_5mC_5hmC_v001.cfg. By our assessment, nanopolish provides more accurate 5-mC estimates than megalodon 2.2.9, which is the only program to provide calls for 5-hmC. In this article, analyses concerning only 5-mC (Figs. 2A, 3A, B, 4B, C, 5A, B) are performed with nanopolish calls and analyses concerning 5-hmC (Figs. 2B, 3C, 4A, 5C, D, 6) are performed with megalodon calls (for both 5-hmC and 5-mC in Fig. 6).

### Analysis of DNA methylation and hydroxymethylation data

Average levels of 5-mC and 5-hmC for each sample were calculated at different genomic annotations defined by chromatin immunoprecipitation sequencing (ChIP-seq), DNase-sequencing, and 15 chromatin states provided by Roadmap Epigenomics project [17] for 27 blood immune cell types (Additional file 1: Table S1). A linear model explaining average methylation on Roadmap annotations was performed for each cell type and annotation separately.

T tests were used to compare average hydroxymethylation change over time in the unaffected mutation carriers against the change in the controls for each annotation separately and for comparing the differences in the proportions of hypermethylated loci at peripheral blood mononuclear cells (PBMCs) enhancers. For transcription factor (TF) binding sites, we used ChIP-seq data from ENCODE [18, 19] GM12878 lymphoblastoid cell line (Additional file 2: Table S2). Linear models were used to model the average methylation on TF binding sites overlapping enhancers.

### Demethylation model

The methylation state changes were modeled with ordinary differential equations in a state model, as shown in Fig. 6A. The model parameters are derived from the equilibrium distribution resulting in *τ* = %C/%5-mC and *ρ* = %C/%5-hmC. The modified base proportions %5-mC and %5-hmC were calculated as mean values over CpG sites at enhancer regions of PBMCs.

### Analysis of RNA-sequencing data

Blood samples for RNA-sequencing were collected using PAXgene Blood RNA tubes (BD biosciences/PreAnalytiX). RNA was extracted from whole blood using PAXgene Blood RNA Kit (Qiagen), and RNA quality was evaluated with TapeStation (Agilent). Sequencing was performed at Macrogen with Illumina 100 bp paired-end reads. Quality and adaptor trimming was done with Trim Galore (v0.5.0) in paired-end mode. Salmon (v0.12.0) was then used for quantification (“quant” mode with --validateMappings) against a human transcriptome index (ensembl release 79). Principal component analysis was run with plotPCA()-function and showed no signs of batch effects (Additional file 1: Figure S1). Variance stabilizing transformation was applied before PCA-plotting. Differential expression analyses in bulk data were run using R (v.4.0.3) packages DESeq2 (v.1.30.0) and tximport (v.1.18.0).

### Deconvolution of bulk RNA-sequencing data

We used deconvolution of the whole blood bulk RNA-sequencing data by CIBERSORTx [20] to estimate the proportions of different blood cell types and to extract their cell-type-specific expression profiles before and after the vitamin C trial. CIBERSORTx utilizes gene expression data from 22 functionally defined human immune cell types (LM22 signature matrix) as a reference in the deconvolution. Before deconvolution, the data were subjected to variance stabilizing transformation in DESeq2. CIBERSORTx was run with bulk-mode batch correction (B-mode) using the LM22 signature matrix provided by the software. For CIBERSORTx high-resolution gene expression profile imputation, cell types from LM22 reference were classified into six groups (B cells, T cells, NK cells, monocytes, granulocytes and other myeloid cells). Differentially expressed genes between unaffected mutation carriers and controls for each cell type at time points at 0 and 12 months were defined using *T* tests and FDR corrections.

For more detailed methods, please see Additional file 1.

## Results

### Epigenetic characteristics of *TET2* mutation carriers

No clear genome-wide differences in DNA methylation levels were present in the data at the beginning of the trial (Fig. 2A). Several studies show that TET2 functions specifically at enhancer regions [21, 22], thus indicating that differences in the average genome-wide methylation levels may not be affected by *TET2* mutation status. The average hydroxymethylation level across the genome was below 5% in all samples; among the mutation carriers, only individuals previously affected by lymphoma showed a slightly lower tendency for 5-hmC levels compared to control individuals (Fig. 2B).

As shown in our previous study, both the wild-type and mutated *TET2* alleles are expressed in the mutation carriers at the mRNA level [12]. *TET2* mRNA expression before the vitamin C trial appeared similar in unaffected individuals with and without *TET2* mutation, whereas individuals previously affected by lymphoma displayed reduced *TET2* expression (Fig. 2C). No statistically significant differences were observed between the unaffected mutation carriers and controls in the expression *TET2* or of other TET genes, although these displayed more variation (Additional file 1: Figure S2).

Using genome annotations provided for 27 blood immune cell types (Additional file 1: Table S1) by the Roadmap Epigenomics project [17], we studied prior to vitamin C supplementation whether *TET2* mutation status had affected methylation levels at different genomic regions in these cell types. Mutation status elevated the average methylation particularly at enhancer regions in multiple cell types (Fig. 3A; Additional file 3: Table S3), supported by earlier studies [21, 22]. The most significant results were obtained from enhancer regions of peripheral blood B cells and PBMCs, of which the latter is highlighted with a red outline in Fig. 3A. Complete results from all cell types are listed in Additional file 3: Table S3. Mutation status also elevated DNA methylation levels at H4K20me1 and H3K4me1 marked chromatin (Additional file 1: Figure S3). These marks have been linked with transcribed regions and the latter particularly with enhancers of differentiation genes in hematopoietic stem cells [23]. Hydroxymethylation levels were not affected by *TET2* mutation status at any specific genomic annotation (Additional file 1: Figure S3, Additional file 3: Table S3). The downstream analyses utilized genome annotations originating from PBMCs, a group broadly representing the different white blood cells and reflecting the results from our linear modeling (Fig. 3A). Average methylation levels showed extensive variation across the genomic annotations, and *TET2* mutant samples showed consistently higher methylation levels at enhancer regions (Fig. 3B). The average hydroxymethylation displayed less variation across the genomic regions (Fig. 3C). One control individual was behaving differently and showing much higher averages on all annotations. This sample had the lowest sequencing quality (Additional file 1: Figure S4), resulting in a low total number of methylation and hydroxymethylation measurements.

### Effect of vitamin C supplementation on DNA methylation and hydroxymethylation

After 12 months of vitamin C intake, the unaffected mutation carriers showed a robust decrease in hydroxymethylation levels (Fig. 4A) (FDR-corrected *p* value < 0.1 for 12/15 tested regions, Welch two-sample *T* test; Additional file 1: Table S4). The phenomenon was visible also in the data collected at 6 months (Additional file 1: Figure S5), whereas no consistent direction of change was present in the methylation data (Fig. 4B and Additional file 1: Figure S5). In our previous study [12], we showed that the family members carrying germline *TET2* mutations had a greater proportion of hypermethylated positions compared to the control family members. Thus, we divided the data into hyper- and hypomethylated loci based on the sample average methylation levels at different genomic regions (see Supplemental Data for detailed methods). Compared to the control individuals, unaffected mutation carriers showed higher proportions of hypermethylated loci at enhancer regions before the trial (Student’s *T* test, *p* = 0.0010, 95% CI [1.52;2.97]%*p*) but not after supplementation with vitamin C for 12 months (Student’s *T* test, *p* = 0.67, 95% CI [− 1.76; 2.47]%*p*) (Fig. 4C and Additional file 1: Figure S6). Thus, vitamin C supplementation seemed to lower the hypermethylation overload previously observed [12] in the mutation carriers.

Studies suggest that *TET2* mutations could predispose to malignancy through methylation-driven disturbance of transcription factor binding [21, 22]. Thus, utilizing ChIP-seq data from the GM12878 lymphoblastoid cell line for 150 transcription factors from the ENCODE project [18, 19] we analyzed the impact of *TET2* mutation status on DNA methylation on TF binding sites. As Ly1 and Ly2 had previously undergone treatment for lymphoma, these individuals were excluded from the analysis in order to identify factors potentially predisposing to lymphomagenesis in the healthy mutation carriers. TET2 functions especially on enhancers, so the analysis was limited to data overlapping these regions. At baseline before vitamin C supplementation, the unaffected mutation carriers displayed higher methylation averages on binding sites of almost all studied transcription factors at enhancers (Additional file 1: Figure S7). We used linear models for each TF, defining average methylation at TF binding sites overlapping enhancers as the response variable, and mutation status together with average enhancer region methylation level as explanatory variables. Using this model, we found no TFs whose binding site methylation level at enhancers would be significantly affected by the mutation status, over and above the generally high methylation level observed at enhancer regions in the mutation carriers (Additional file 1: Figure S8). Next, we further limited our enhancer region analysis to sites showing sample-specific hypermethylation: Only positions showing higher methylation than the sample’s average methylation at enhancers were included to specifically identify binding sites with hypermethylation overload. Before vitamin C supplementation, *TET2* mutation status was associated with significant binding site hypermethylation of multiple transcription factors at enhancers (Fig. 5A; Additional file 4: Table S5). These included the master hematopoietic regulators SPI1 (PU.1) and TBX21, as well as ETS family TF ELF1 reported also in our earlier study [12]. After 12 months of vitamin C supplementation, mutation status no longer showed a significant effect on TF binding site methylation for any of the studied factors (Fig. 5B).

As the *TET2* mutation status did not have an effect on the hydroxymethylation levels at any specific genomic annotation (Additional file 1: Figure S3), we performed the TF binding site analysis using genome-wide hydroxymethylation data. In this analysis, only the binding sites of REST showed significantly higher hydroxymethylation values in the mutation carriers at the beginning of the trial (Fig. 5C). After 12 months of vitamin C supplementation, mutation carriers displayed elevated hydroxymethylation levels at binding sites of TBX21 (*p* = 0.0291), but this remained nonsignificant after correction for multiple testing (Fig. 5D).

### Changes in gene expression

Differential expression analysis comparing the unaffected mutation carriers to the control individuals before the vitamin C supplementation yielded 35 differentially expressed (DE) genes (Additional file 5: Table S6). PANTHER overrepresentation test against the Reactome pathway database showed modest enrichment on Hemostasis (R-HSA-109582: *GP1BB*, *PF4*, *PPBP*, *PRKAR2B*, *THBS1*, *TUBB1*) (*p*-adj. = 4.02*10^−2^) for genes downregulated (*n* = 19) in the mutation carriers. The upregulated genes (*n* = 16) showed no statistically significant enrichment on any pathways. After 12 months of vitamin C intake, the number of differentially expressed genes decreased from the 35 genes detected at baseline to 16 genes. None of the hemostasis-related genes differentially expressed at baseline remained differentially expressed after the vitamin C trial. As vitamin C transport to cells is mostly controlled by *SLC23A1* and *SLC23A2* [24]*,* we visualized the expression of these genes separately (Additional file 1: Figure S9). No clear differences were present in the expression of these genes before or after the vitamin C trial. As expected, *TET2* showed no significant expression differences before or after the trial between the unaffected mutation carriers and controls.

We used the whole blood bulk RNA-sequencing data to estimate the proportions of blood cell types in each individual. CIBERSORTx [20] deconvolution results reflected the expected distribution of different cell types in blood: Neutrophils were the most common cell type followed by monocytes and CD4+ T cells, while dendritic cells were rare at < 1% and macrophages absent (Additional file 1: Figure S10). As expected, we observed some variations in blood cell-type proportions among the study individuals, but no clear differences between the mutation carriers and controls (Additional file 1: Figure S10). The estimated cell-type proportions were similar before and after the vitamin C trial (Additional file 1: Figure S11). In line with these data, our previous study [12] showed that blood leukocyte counts of the *TET2* mutation carriers were grossly normal, and only further detailed T- and B-lymphocyte subtyping analyses showed consistent changes compared to reference values in certain subpopulations. Cell-type-specific expression profiles from CIBERSORTx revealed no differentially expressed genes between the mutation carriers and controls either before or after the vitamin C trial (Additional file 6: Table S7).

### DNA methylation turnover

To reconcile the changes observed in both 5-mC and 5-hmC at enhancer regions, we combined DNA methylation and demethylation in a state-space model shown in Fig. 6A. We assume that an unmethylated CpG site (state C in Fig. 6A) is methylated with a constant unit rate to 5-mC, which is oxidized to 5-hmC by TET2 with rate τ, followed by further TET2-mediated oxidation to 5-fC and 5-caC that can be actively excised and ultimately repaired back to unmethylated C with an overall repair rate ρ. These latter steps involved in active demethylation require the activity of TET2 and several other enzymes (Fig. 1C). Fitting the observed 5-mC and 5-hmC percentages to the resulting equilibrium distribution provides point estimates for the rates *τ* and *ρ*. On PBMC enhancer regions, the TET2 mutation carriers show a clear increase in ρ rate after 12 months of vitamin C supplementation (Fig. 6B; Additional file 1: Table S8). When analyzing data from both 6- and 12-month time points, kernel density estimates revealed consistently increased *ρ* in the mutation carriers, while the estimates for control individuals are spread around the origo suggesting no uniform effect (Additional file 1: Figure S12). Changes in *τ* are smaller and less consistent between the samples (Fig. 6B; Additional file 1: Figure S12).

The differential response to vitamin C in TET2 mutant individuals in ρ and not *τ* is consistent with a model where TET2 remains bound to oxidized 5-hmC and vitamin C reduces Fe^3+^–Fe^2+^, enabling further oxidation in a single binding event of TET2 [5, 7, 25]. The initial binding and first oxidation of 5-mC to 5-hmC (modeled with *τ*) might be a rate-limiting step in *TET2* mutation carriers but not in individuals with two working copies of TET2. Thus, in TET2 mutant individuals, vitamin C increases the turnover rate of 5-mC mostly by driving the cytosine oxidation cascade to completion at a higher overall rate. However, as individual variation in intracellular vitamin C levels may have existed at the beginning of the trial, the results of this modeling must be interpreted with caution.

## Discussion

We report here the long-term effects of dietary supplementation with 1 g of vitamin C on methylation, hydroxymethylation, and gene expression in individuals carrying a germline loss-of-function mutation in *TET2*. These individuals display hypermethylation overload in blood, predisposing them to an increased risk of developing lymphoma [12]. Here, we found a significantly higher proportion of hypermethylated positions at enhancer regions in the unaffected mutation carriers before the trial, but not after 12 months of vitamin C supplementation. Compatible with previous studies [12, 26], the binding sites of key hematopoietic regulators such as SPI1 (PU.1), TBX21 and ELF1 were hypermethylated at enhancer regions in the *TET2* mutation carriers before vitamin C supplementation. Hypermethylation at the binding sites of these and most of the other examined transcription factors was shown to be reversed by vitamin C supplementation. These data suggest that vitamin C alleviates the methylation defect observed at enhancer regions. The result is promising as TET2-mediated demethylation specifically at enhancer regions is known to play important roles in development, cell differentiation, and hematological disease [21, 22, 26–29]. While we believe that the effects of vitamin C seen in this study are mediated by improved TET2 function, the precise mechanism of action and protein level interactions between all the members of the demethylation cascade and vitamin C should be studied further.

Unexpectedly, vitamin C supplementation also resulted in decreased genomic hydroxymethylation in the unaffected mutation carriers. This seemed to contradict studies in cultured cells where hydroxymethylation levels were increased in response to vitamin C [5, 10, 30]. However, these studies examined short-term vitamin C treatment in the range of hours to days. Even at these time scales, it was noted that the rapid increase in 5-hmC levels in response to vitamin C was only transient, while 5-mC levels continued to decrease [10, 30]. Notably, in embryonic stem cells, levels of 5-hmC returned to baseline or even below it at 72 h of vitamin C treatment; the authors state that this effect may be explained by the loss of 5-mC substrate as a result of demethylation [10]. Thus, our observations in mixed blood cells after 6–12 months of vitamin C supplementation in vivo likely reflect a new steady state in the system after a transient 5-hmC induction, highlighting the more persistent effects of vitamin C on the epigenome.

TET enzymes catalyze multiple reaction steps and collaborate with other enzymes to complete the demethylation cycle and restore unmodified cytosine. Thus, the hydroxymethylation levels alone do not fully depict TET activity nor the further TDG/BER-mediated reaction steps involved in replication-independent active demethylation. As such, 5-hmC does not lead to active demethylation and a substantial proportion undergoes replication-dependent dilution [31]; only the further TET oxidation products 5-fC and 5-caC are substrates for TDG. Importantly, in mouse embryonic stem cells, 5-fC and 5-caC are more potently induced by vitamin C compared to 5-hmC [5], and accumulation of unmodified C in response to vitamin C specifically requires the TDG/BER-mediated 5-fC/5-caC excision steps [6]. These data suggest that 5-fC and 5-caC are the main targets for vitamin C effects in DNA demethylation.

Using long-read nanopore sequencing combined with methylation calling [32], we were able to directly detect both 5-mC and 5-hmC modifications at the level of single DNA molecules, in contrast to bisulfite sequencing that cannot distinguish between the two and relies on indirect detection via base conversion. While this provides us with high-quality data on both DNA methylation and demethylation, there are currently no tools to call 5-fC and 5-caC modifications from nanopore data. We thus turned to modeling of the reaction rates involved in DNA methylation/demethylation. The results from the modeling suggested that vitamin C may increase the turnover rate of 5-mC when a limiting factor, such as the reduced amount of functional TET2 molecules in the mutation carriers, is present. Under such conditions, vitamin C specifically enhanced the rate of the later oxidation (TET2) and repair (TDG/BER) steps converting 5-hmC to unmodified cytosine, while the rate of the first oxidation step (5-mC to 5-hmC) did not show consistent changes.

Interestingly, TET2 can yield 5-fC and 5-caC iteratively in a single encounter with 5-mC containing DNA, without releasing a 5-hmC intermediate [25]. Enhancement of such a mode of action by vitamin C would be compatible with our observations from the modeling of reaction rates in the methylation/demethylation cycle. A conserved active site threonine in human TET2 has been identified as a crucial determinant required for 5-fC and 5-caC generation via the identification of base substitutions that cause stalling of oxidation at 5-hmC [33]. As vitamin C exhibits strong binding to the TET2 catalytic domain, more strongly induces 5-fC and 5-caC compared to 5-hmC and requires TDG to induce unmodified C [5–7], effects on TET2 processivity seem possible yet these have not been directly addressed. A recent study utilizing the TET mutants stalling oxidation at 5-hmC showed that only TET enzymes proficient for oxidation to 5-fC/5-caC can rescue the reprogramming potential of *Tet2*-deficient somatic cells to pluripotency by inducing rapid DNA demethylation at reprogramming enhancers [34]. Thus, despite the low prevalence of 5-fC/5-caC sites in the genome, these TET2 oxidation products can act as major drivers of DNA demethylation and changes in their production rates at enhancers can have profound biological effects.

As expected, vitamin C supplementation resulted in changes at DNA methylation and hydroxymethylation level rather than causing extensive transcriptional changes. However, we observed a decreased number of differentially expressed genes between the healthy *TET2* mutation carriers and control individuals after vitamin C supplementation, though expression differences were modest also at baseline.

Limitations of the study include a small sample size, precluding the use of more sophisticated statistical models, such as models accounting for age and sex. Our cytosine demethylation model ignores the cell cycle, and our data allow only steady-state observations. Baseline plasma or intracellular levels of vitamin C were not measured, introducing unknown individual variations. In contrast to the unaffected mutation carriers, two mutation carriers previously diagnosed with lymphoma did not show vitamin C-driven methylation changes. Older age, chemotherapy, and in particular the low baseline expression of *TET2* compared to other study participants may have contributed. Thus, the boost of 1 g of vitamin C may not be sufficient to rescue TET2 function in these individuals. The same may be true for doses smaller than 1 g, as the individual who had taken 580 mg daily already prior to the study start did not differ from others in the baseline analyses.

## Conclusions

The results on the effect of vitamin C on DNA methylation turnover are promising. Some studies have reported the effects of vitamin C in vivo in patients with hematological malignancy, focusing on the potential of vitamin C in treatment [35, 36]. Our results provide additional rationale for such efforts. Furthermore, our results suggest that vitamin C may be of value in the prevention of hematological neoplasia for many individuals carrying germline [12, 37–39] or somatic *TET2* mutations. Further studies are warranted to confirm our molecular findings as well as to examine the putative value of vitamin C supplementation in the prevention of TET2-related hematological neoplasia.

## Supplementary Information

**Additional file 1: Supplemental Data.** Supplemental Methods, Supplemental Tables 1,4 and 8, Supplemental Figures and descriptions for additional files 2–6.

**Additional file 2: Supplemental Table 2.** List of transcription factor names and ENCODE file accession IDs.

**Additional file 3: Supplemental Table 3**. Results from linear models explaining average methylation and hydroxymethylation at different genome annotations from the Roadmap Epigenomics project.

**Additional file 4: Supplemental Table 5.** Results from transcription factor binding site methylation analysis.

**Additional file 5: Supplemental Table 6.** Results from gene expression analyses.

**Additional file 6: Supplemental Table 7.** Cell type specific expression differences between unaffected mutation carriers and controls.

## Data Availability

Processed methylation and hydroxymethylation data used as inputs for the analyses as well as processed RNA sequencing data have been deposited at the European Genome–phenome Archive (EGA; https://www.ebi.ac.uk/ega/) under accession number EGAS00001006916. A data access committee has been established to ensure that the intended use of data is compatible with the requirements of the European General Data Protection Regulation (GDPR) and consistent with the consents given. R-scripts used to generate methylation and hydroxymethylation plots are available at Zenodo (zenodo.org, 10.5281/zenodo.7500493). The input for the demethylation model is provided in Additional file 1: Table S8. Results from differential expression analysis are provided in Additional file 5: Table S6 and Additional file 6: Table S7.

## Abbreviations

AML: Acute myeloid leukemia
TET: Ten–eleven translocation
5-mC: 5-Methylcytosine
5-hmC: 5-Hydroxymethylcytosine
5-fC: 5-Formylcytosine
5-caC: 5-Carboxylcytosine
Gulo: Gulonolactone oxidase
NLPHL: Nodular lymphocyte-predominant Hodgkin lymphoma
DNMT: DNA methyltransferase
α-KG: α-Ketoglutarate
TDG: Thymine DNA glycosylase
BER: Base excision repair
Chip-seq: Chromatin immunoprecipitation sequencing
PBMC: Peripheral blood mononuclear cell
TF: Transcription factor
PCA: Principal component analysis
DE: Differentially expressed
