# Supplementary material for: Vitamin C boosts DNA demethylation in TET2 germline mutation carriers
Source: Clin Epigenetics. 2023 Jan 14;15:7. doi: 10.1186/s13148-022-01404-6 (PMC9840351; doi:10.1186/s13148-022-01404-6)
Supplement: Supplementary file 1 — Additional file 1: Supplemental Data. Supplemental Methods, Supplemental Tables 1,4 and 8, Supplemental Figures and descriptions for additional files 2–6. [file 13148_2022_1404_MOESM1_ESM.pdf]

Supplemental Data

Supplemental Methods **3**

Identification of germline variants in TET2 3

Analysis of DNA methylation and hydroxymethylation data 3

Demethylation model 5

Differential expression analyses 5

Supplemental Table 1. Roadmap cell types and annotations used in the analyses. 7

Supplemental Table 2. List of transcription factor names and ENCODE file accession IDs. 8

Supplemental Table 3. Results from linear models explaining average methylation and hydroxymethylation at different genome annotations from the Roadmap Epigenomics project. 9

Supplemental Table 4. Results from the hydroxymethylation difference analysis. 10

Supplemental Table 5. Results from transcription factor binding site methylation analysis. 11

Supplemental Table 6. Results from gene expression analyses. 12

Supplemental Table 7. Cell type specific expression differences between unaffected mutation carriers and controls. 13

Supplemental Table 8. Parameters for demethylation model. 14

Supplemental Figure 1. Principal component analysis of the bulk RNA-sequencing data. 15

Supplemental Figure 2. Expression of TET enzymes at baseline. 16

Supplemental Figure 3. Effect of TET2 germline mutation on hydroxymethylation and methylation at different genomic annotations. 17

Supplemental Figure 4. Quality metrics for Promethion data. 19

Supplemental Figure 5. Average change in genomic methylation and hydroxymethylation levels in control and TET2 mutant individuals after 6 months of vitamin C supplementation. 20

Supplemental Figure 6. Proportion of hypermethylated positions at PBMC annotations at different time points. 21

Supplemental Figure 7. Average methylation values at different transcription factor binding sites overlapping PBMC enhancers before vitamin C supplementation. 22

Supplemental Figure 8. Effect of TET2 mutation on methylation at transcription factor binding sites overlapping enhancers. 23

Supplemental Figure 9. Expression of SLC23 ascorbate transporters. 24

Supplemental Figure 10. Estimated proportions of cell types in blood from deconvolution of RNA-sequencing data. 25

Supplementary Figure 11. Changes in estimated cell type proportions after the vitamin C trial. 26

Supplemental Figure 12. Demethylation model parameter changes in 6 and 12 month timepoints. 27

References for Supplemental Data 28

Supplemental Methods

Identification of germline variants in *TET2*

Analysis of the germline *TET2* NM_001127208.2:c.4500delA variants segregating with lymphoma is described in our previous publication on this family[1].

Analysis of DNA methylation and hydroxymethylation data

All analyses included only autosomal CpGs with read coverage of 3-75. We used R (R v.4.1.2; tidyverse v.1.3.0; ggrepel 0.9.1; ggpubr 0.4.0) for visualization of the 5-mC and 5-hmC data. The 15 chromatin state annotation used for the average 5-mC (nanopolish) and 5-hmC (megalodon) calculation for each sample was provided by Roadmap Epigenomics project[2] and was based on Chromatin immunoprecipitation sequencing (Chip-seq), DNase-sequencing data for 27 blood immune cell types (**Supplemental Table 1**). For transcription factor binding sites we used Chip-seq data from the ENCODE GM12878 lymphoblastoid cell line[3,4]: 182 binding site datasets covering 150 transcription factors (TFs) entered into the analyses (**Supplemental Table 2**). A linear model explaining the average methylation on Roadmap annotations were performed for each cell type and annotation separately. The average methylation (or hydroxymethylation) values on annotations per sample served as response variables while *TET2* mutation status, lymphoma status, and genome wide average methylation values served as explanatory variables (lm(average methylation ~ mutation status + lymphoma status + genome wide average methylation)). Coefficient estimates and p-values related to *TET2* mutation status were collected and visualized as volcano plots using R. In the TF binding site analyses Ly1&Ly2 were excluded. For methylation data, only CpG positions overlapping with PBMC enhancers were included into analyses and a model lm(average methylation on TF binding sites ~ mutation status + average methylation on PBMC enhancers) was used for each TF

data set separately. In the second analysis, only hypermethylated positions were included when calculating the average methylation at TF binding sites. To define hypermethylated positions, we first calculated the average methylation value at PBMC enhancers for each sample separately. Subsequently, we defined for each sample the enhancer positions having higher methylation value than the sample's average enhancer region methylation. These positions were called as hypermethylated.

As the *TET2* mutation status did not have an effect on hydroxymethylation levels at any specific genomic annotation (**Supplemental Figure 3**), we performed the TF binding site analysis using genome-wide hydroxymethylation data. For hydroxymethylation data, a model lm(average hydroxymethylation on TF binding sites ~ mutation status + average hydroxymethylation across all TF binding sites) was used. All linear models were run for baseline (0 months) and 12 months data separately.

For the average methylation and hydroxymethylation change between time points for each individual on different annotation regions, we first calculated the difference in 5-(h)mC for each site and then calculated the mean difference for sites in each annotation. T-test with unequal variances was used to compare average hydroxymethylation change (0 months vs 12 months) in the unaffected mutation carriers compared to that in the controls for each annotation separately.

To test the difference in proportion of hypermethylated loci at enhancer regions between the unaffected mutation carriers and control individuals, we first defined the hypermethylated positions at PBMC enhancers (see above). Next, we defined the proportion of hypermethylated loci at enhancer regions for each sample. T-test with equal variances was used to compare the differences in the proportions between the unaffected mutation carriers and the control individuals first at baseline (0 months) and then at 12 months. Only the same

positions having read coverage at both timepoints (0/6 months, or 0/12 months) were included in visualization and testing.

Demethylation model

The model has three possible states for a CpG site: C, 5-mC or 5-hmC. The state changes, strictly following the cycle C -> 5mC -> 5hmC -> C, are modeled as Ordinary Differential Equations (**Main Figure 6A**). The model parameters are derived from the equilibrium distribution resulting in τ=%C/%5mC and ρ=%C/%5hmC. The modified base proportions %5mC and %5hmC were calculated as mean values over CpG sites at enhancer regions in PBMCs covered with 3-75 reads. The percentage of unmethylated C is calculated as %C= 100-%5mC-%5hmC, which is consistent with unmodified C call from megalodon. In this analysis, both the methylation and hydroxymethylation calls were done with megalodon. This model has some nonobvious limitations: Firstly, it ignores the cell cycle and associated maintenance or dilution of methylation and hydroxymethylation, and hence assumes that all demethylation happens actively through the TET2/TDG/BER pathway. Secondly, each model parameter comprises a long cascade of reactions that are likely under complex, unmodelled regulation. Thirdly, our data allows only steady state observations of the model, as our 6 month and 12 month timepoints are late considering the estimated lifespans of white blood cells ranging from a few days (neutrophils, monocytes) to 60-320 days estimated for mature lymphocytes[5]. Despite being a limitation in this model, the steady state is an outcome of interest from a clinical point of view where persistent long-term effects of vitamin C are of significant interest.

Differential expression analyses

Differential expression analyses were run using R (v.4.0.3) packages DESeq2 (v.1.30.0) and tximport (v.1.18.0). For all analyses, we applied a minimum filtering of 10 counts per gene

over all samples. Gene expression differences between the unaffected mutation carriers and control individuals at baseline and at 12 months were defined with DESeq()-function likelihood ratio test (LRT) with a design of (expression = ~RIN + TET2 mutation status) and with a reduced model (expression = ~RIN). Statistical overrepresentation test for genes up- and downregulated in the mutation carriers was run using PANTHER[6] (http://pantherdb.org) Overrepresentation Test (Released 20220202) against Reactome database (Reactome version 65 Released 2020-11-17) using Fisher's exact test with FDR correction. Visualizations were performed using plotCounts() -function for data imported with DESeqDataSetFromTximport() without specifying the design model.

Supplemental Table 1. Roadmap cell types and annotations used in the analyses.

Roadmap ID	Cell type
E029	CD14_Primary_Cells
E030	CD15_Primary_Cells
E031	CD19_Primary_Cells_Cord_Bl
E032	CD19_Primary_Cells_Peripheral_UW
E033	CD3_Primary_Cells_Cord_Bl
E034	CD3_Primary_Cells_Peripheral_UW
E035	CD34_Primary_Cells
E036	CD34_Cultured_Cells
E037	CD4_Memory_Primary_Cells
E038	CD4_Naive_Primary_Cells
E039	CD4+_CD25-_CD45RA+_Naive_Primary_Cells
E040	CD4+_CD25-_CD45RO+_Memory_Primary_Cells
E041	CD4+_CD25-_IL17-_PMA-Ionomycin_stimulated_MACS_purified_Th_Primary_Cells
E042	CD4+_CD25-_IL17+_PMA-Ionomcyin_stimulated_Th17_Primary_Cells
E043	CD4+_CD25-_Th_Primary_Cells
E044	CD4+_CD25+_CD127-_Treg_Primary_Cells
E045	CD4+_CD25int_CD127+_Tmem_Primary_Cells
E046	CD56_Primary_Cells
E047	CD8_Naive_Primary_Cells
E048	CD8_Memory_Primary_Cells
E050	Mobilized_CD34_Primary_Cells_Female
E051	Mobilized_CD34_Primary_Cells_Male
E062	Peripheral_Blood_Mononuclear_Primary_Cells
E115	Dnd41_TCell_Leukemia
E116	GM12878_Lymphoblastoid
E123	K562_Leukemia
E124	Monocytes-CD14+_RO01746

Abbreviation	Chromatin state
TssA	Active TSS
TssAFlnk	Flanking Active TSS
TxFlnk	Transcr. at gene 5' and 3'
Tx	Strong transcription
TxWk	Weak transcription
EnhG	Genic enhancers
Enh	Enhancers
ZNF/Rpts	ZNF genes & repeats
Het	Heterochromatin
TssBiv	Bivalent/Poised TSS
BivFlnk	Flanking bivalent TSS/Enh
EnhBiv	Bivalent enhancer
ReprPC	Repressed polycomb
ReprPCWk	Weak repressed polycomb
Quies	Quiesent/Low

List of histone marks used in the analyses:

DNase
H3K27ac
H3K27me3
H3K36me3
H3K4me1
H3K4me3
H3K9me3
H3K9ac
H2A.Z
H3K4me2
H3K79me2
H4K20me1
H3K9me1

Supplemental Table 2. List of transcription factor names and ENCODE file accession IDs.

This large Supplemental table can be downloaded separately. The table contains the list of transcription factors used in the analyses and the corresponding ENCODE file Ids.

Supplemental Table 3. Results from linear models explaining average methylation and hydroxymethylation at different genome annotations from the Roadmap Epigenomics project.

This large Supplemental table can be downloaded separately. The table contains detailed statistics from the linear modeling results.

Supplemental Table 4. Results from the hydroxymethylation difference analysis.

T-test with unequal variances was used to compare average 5-hmC change (0 months vs 12 months) in the unaffected mutation carriers against the change of the controls for each annotation separately.

p_value	annotation	CI	FDR_p_value
0,001746504	BivFlnk	-0.504741619606759;-0.230365700393241	0,01309878
0,001126639	EnhBiv	-0.720271424503313;-0.391715908830021	0,01309878
0,004597299	TssA	-0.618209892654916;-0.225394507345084	0,022986496
0,012673935	TssAFlnk	-0.771449300885359;-0.202825965781308	0,047527255
0,022803701	TssBiv	-0.546040362553501;-0.071788304113166	0,05543028
0,018895675	ReprPC	-1.37080415422476;-0.30623651244191	0,05543028
0,025867464	Enh	-2.281022262478;-0.332423737521999	0,05543028
0,04396341	ReprPCWk	-3.06404958713699;-0.0894664128630088	0,060831505
0,038513234	Quies	-3.54131364073599;-0.187679692597342	0,060831505
0,04460977	Tx	-2.41655103338967;-0.0623722999436614	0,060831505
0,042666677	EnhG	-2.58451876069786;-0.101823905968805	0,060831505
0,049466263	TxWk	-3.02232710260917;-0.0073075640574966	0,061832828
0,088208602	Het	-4.28408258803884;0.602523921372178	0,101779156
0,141604211	TxFlnk	-2.66720575801935;0.733947024686014	0,151718797
0,155825098	ZNF/Rpts	-5.08105576209316;1.53989042875983	0,155825098

Supplemental Table 5. Results from transcription factor binding site methylation analysis.

This large Supplemental table can be downloaded separately.Table contains the results from linear models explaining the average methylation values on transcription factor binding sites before and after vitamin C trial.

Supplemental Table 6. Results from gene expression analyses.

This large Supplemental table can be downloaded separately. The table contains the results from differential expression analysis comparing healthy mutation carriers to control individuals at baseline (0 months) and after 12 months intake of vitamin C (12 months).

Supplemental Table 7. Cell type specific expression differences between unaffected mutation carriers and controls.

This large Supplemental table can be downloaded separately. The table contains the results from differential expression analysis comparing healthy mutation carriers to control individuals at baseline (0 months) and after 12 months intake of vitamin C (12 months) in deconvoluted cell types.

Supplemental Table 8. Parameters for demethylation model.

sample	time	nMeasurements	5-mC average	5-hmC average	mut Status	REPAIR rate	TET rate
**Ly11**	**0_months**	507857	63.698	3.254	MUT	10.1556	0.5188
	**6_months**	422532	64.600	2.070	MUT	16.1014	0.5159
	**12_months**	351907	68.095	2.517	MUT	11.6777	0.4316
**Ly1**	**0_months**	602427	63.978	1.662	Ly_Mut	20.6726	0.5371
	**6_months**	604639	62.655	2.044	Ly_Mut	17.2667	0.5634
	**12_months**	468912	66.413	1.947	Ly_Mut	16.2530	0.4764
**Ly1311**	**0_months**	139810	63.718	4.365	Control	7.3123	0.5009
	**6_months**	645812	62.536	3.073	Control	11.1903	0.5499
	**12_months**	75755	62.497	5.925	Control	5.3293	0.5053
**Ly1313**	**0_months**	545837	59.826	2.541	Control	14.8085	0.6290
	**6_months**	289231	62.738	4.397	Control	7.4739	0.5238
	**12_months**	191739	63.358	2.692	Control	12.6113	0.5358
**Ly1314**	**0_months**	447201	62.785	2.963	Control	11.5606	0.5455
	**6_months**	577466	60.132	3.006	Control	12.2646	0.6130
	**12_months**	530434	60.907	3.207	Control	11.1893	0.5892
**Ly1315**	**0_months**	678866	61.481	3.438	MUT	10.2051	0.5706
	**6_months**	589896	62.373	1.939	MUT	18.4044	0.5722
	**12_months**	362923	63.660	2.073	MUT	16.5294	0.5383
**Ly2**	**0_months**	680646	66.170	2.347	Ly_Mut	13.4161	0.4758
	**6_months**	725393	62.306	1.705	Ly_Mut	21.1069	0.5776
	**12_months**	333364	62.534	1.887	Ly_Mut	18.8532	0.5689
**Ly9**	**0_months**	390681	67.694	3.310	MUT	8.7592	0.4283
	**6_months**	663310	63.680	2.437	MUT	13.9010	0.5321
	**12_months**	583778	63.854	2.623	MUT	12.7804	0.5250

Number of observed CpG sites, methylation and hydroxymethylation percentage, mutation status and Repair rate ρ, and TET rate τ for samples on PBMC Enhancer regions.

Supplemental Figure 1. Principal component analysis of the bulk RNA-sequencing data.

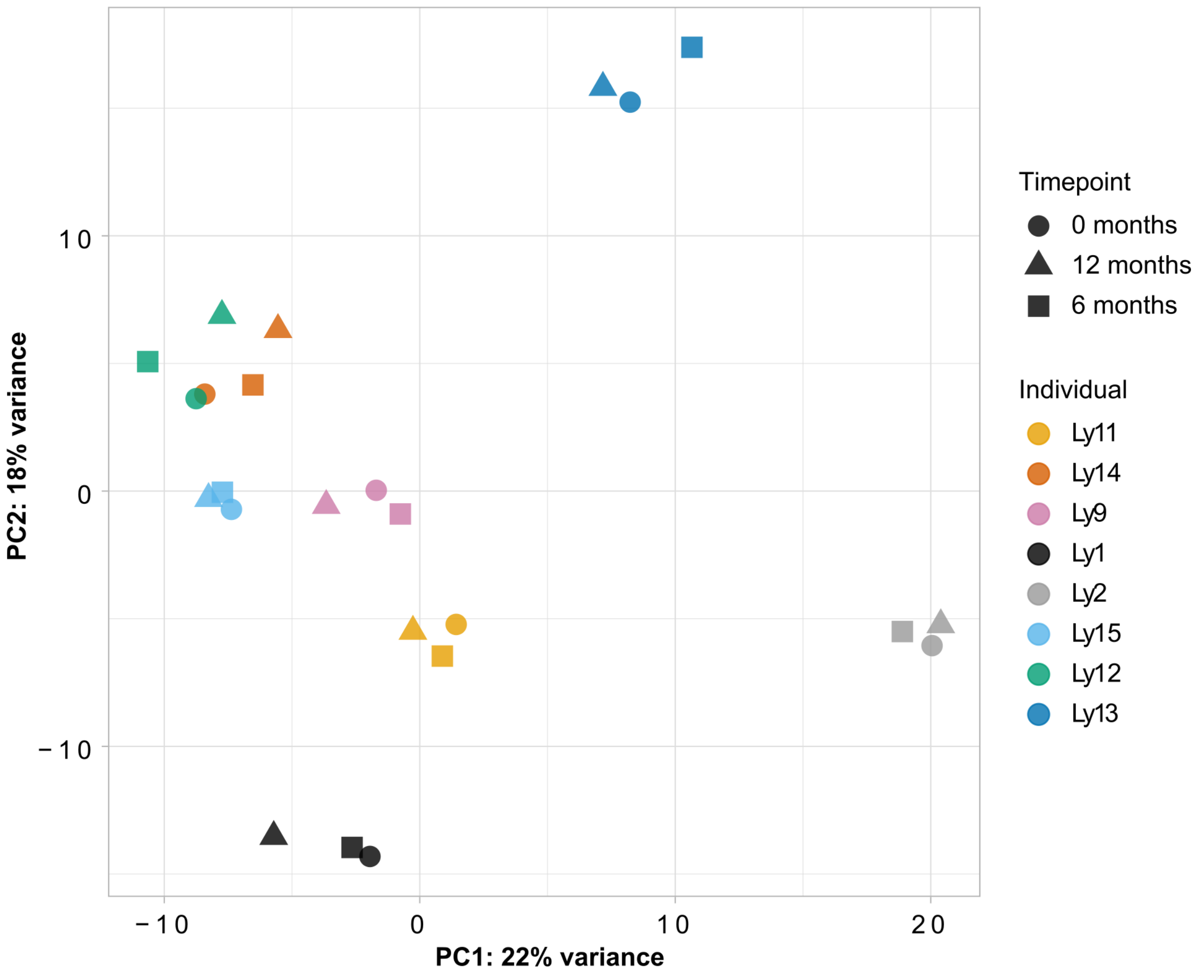

PCA-plot of the RNA-sequencing data showed no signs of batch effects depending on the time point.

Supplemental Figure 2. Expression of TET enzymes at baseline.

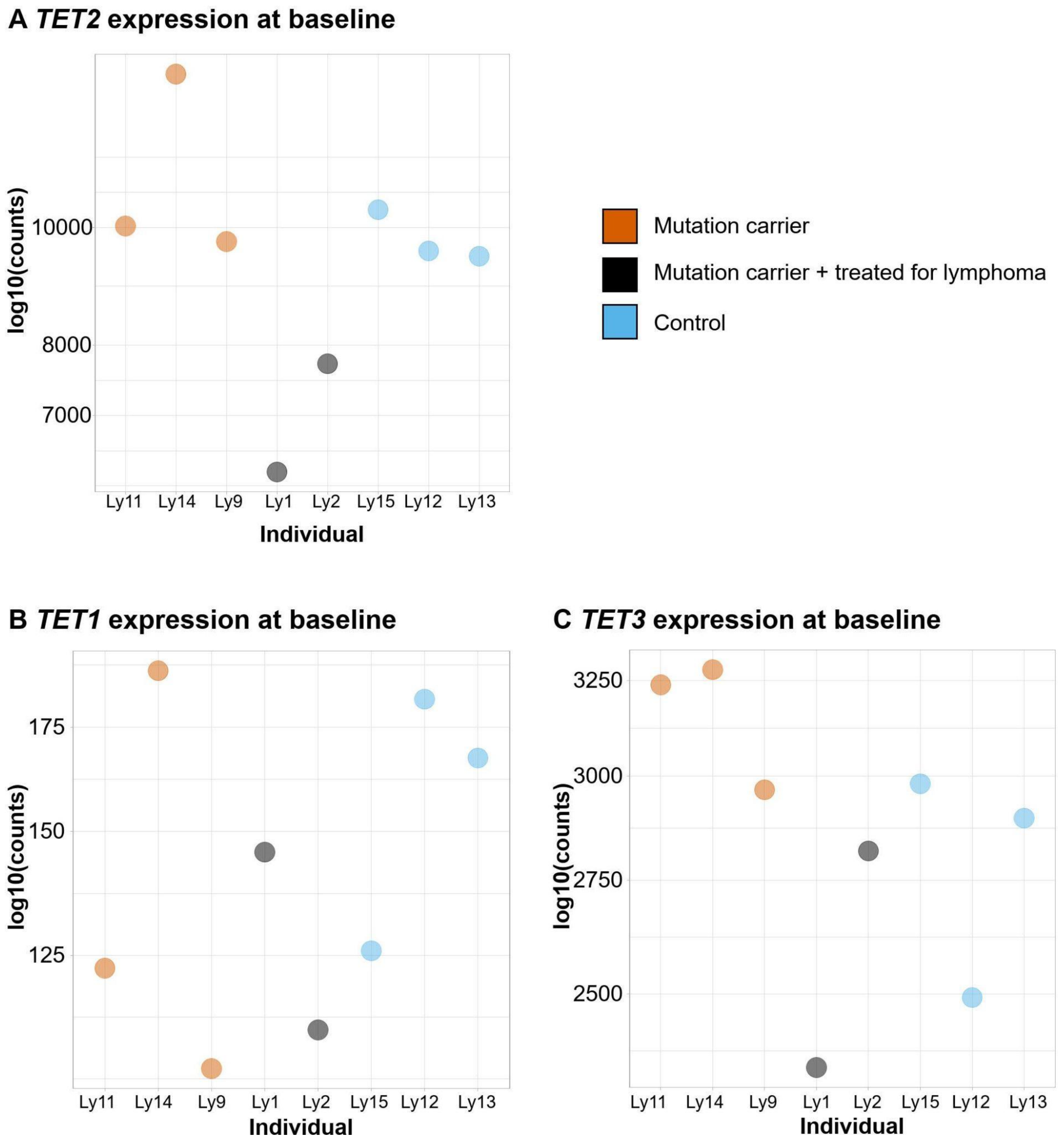

(A)*TET2* gene expression from RNA-seq at baseline before vitamin C supplementation (0 months). **(B)** *TET1* showed lower expression in two unaffected mutation carriers compared to the control individuals at baseline. **(C)** *TET3* expression was higher in two unaffected mutation carriers compared to the control individuals at baseline.

Supplemental Figure 3. Effect of TET2 germline mutation on hydroxymethylation and methylation at different genomic annotations.

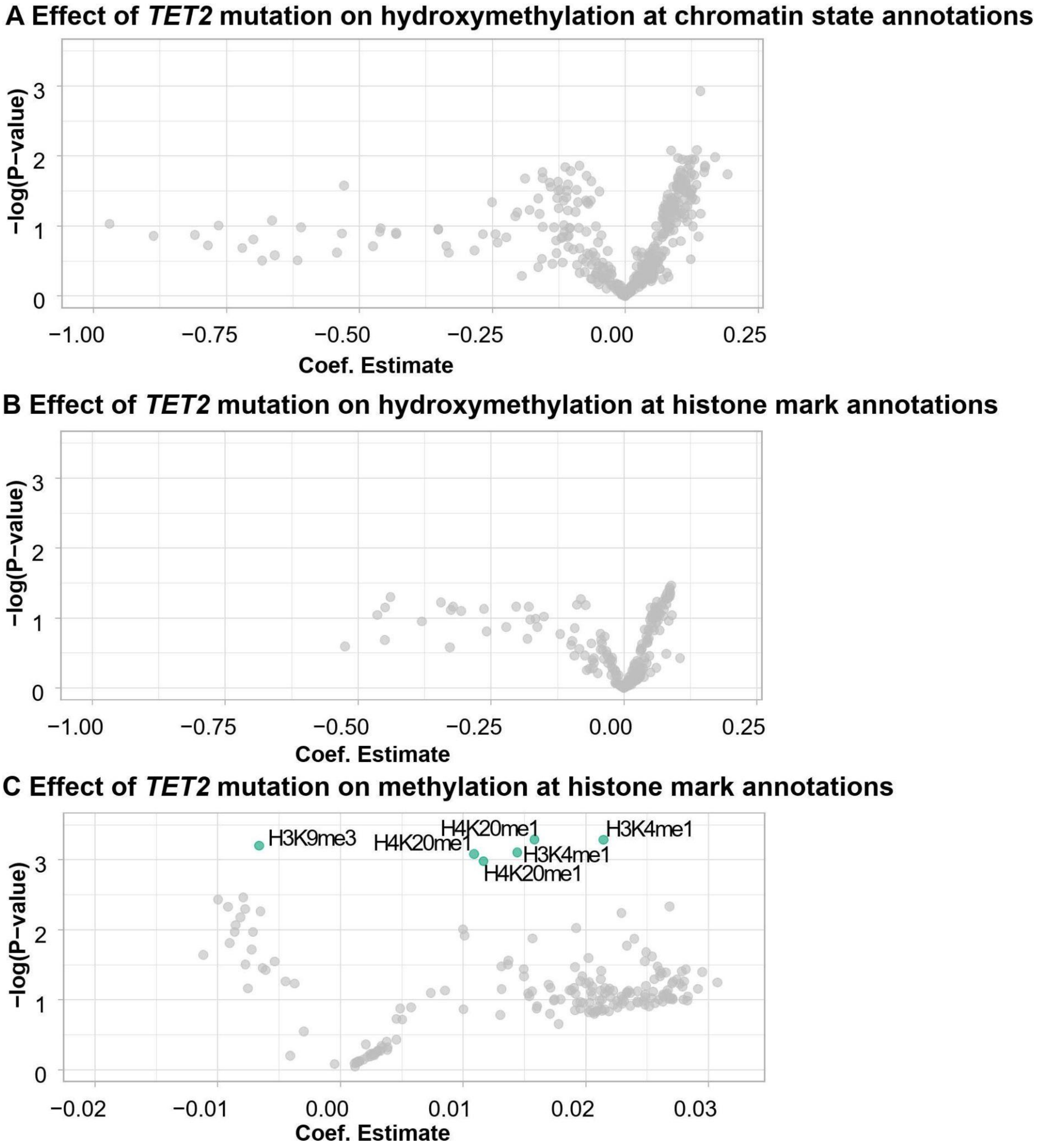

(A) Volcano plot showing the effect of *TET2* mutation status on average hydroxymethylation in different cell types and chromatin state annotations and **(B)** at histone mark annotations. Model lm(average hydroxymethylation ~ mutation status + lymphoma status + genome wide average hydroxymethylation) was fitted over each cell type and annotation separately. Each point represents results related to mutation status from one cell type and annotation. Mutation status did not have an effect on the average hydroxymethylation levels at any

genomic annotation across different cell types. **(C)** Volcano plot showing the effect of *TET2* mutation status on average methylation in different cell types and histone mark annotations. Please note the different X-axis scaling in C. Model lm(average methylation ~ mutation status + lymphoma status + genome wide average methylation) was fitted over each cell type and annotation separately. Mutation carriers displayed elevated DNA methylation levels at H4K20me1 and H3K4me1 marked chromatin.

Supplemental Figure 4. Quality metrics for Promethion data.

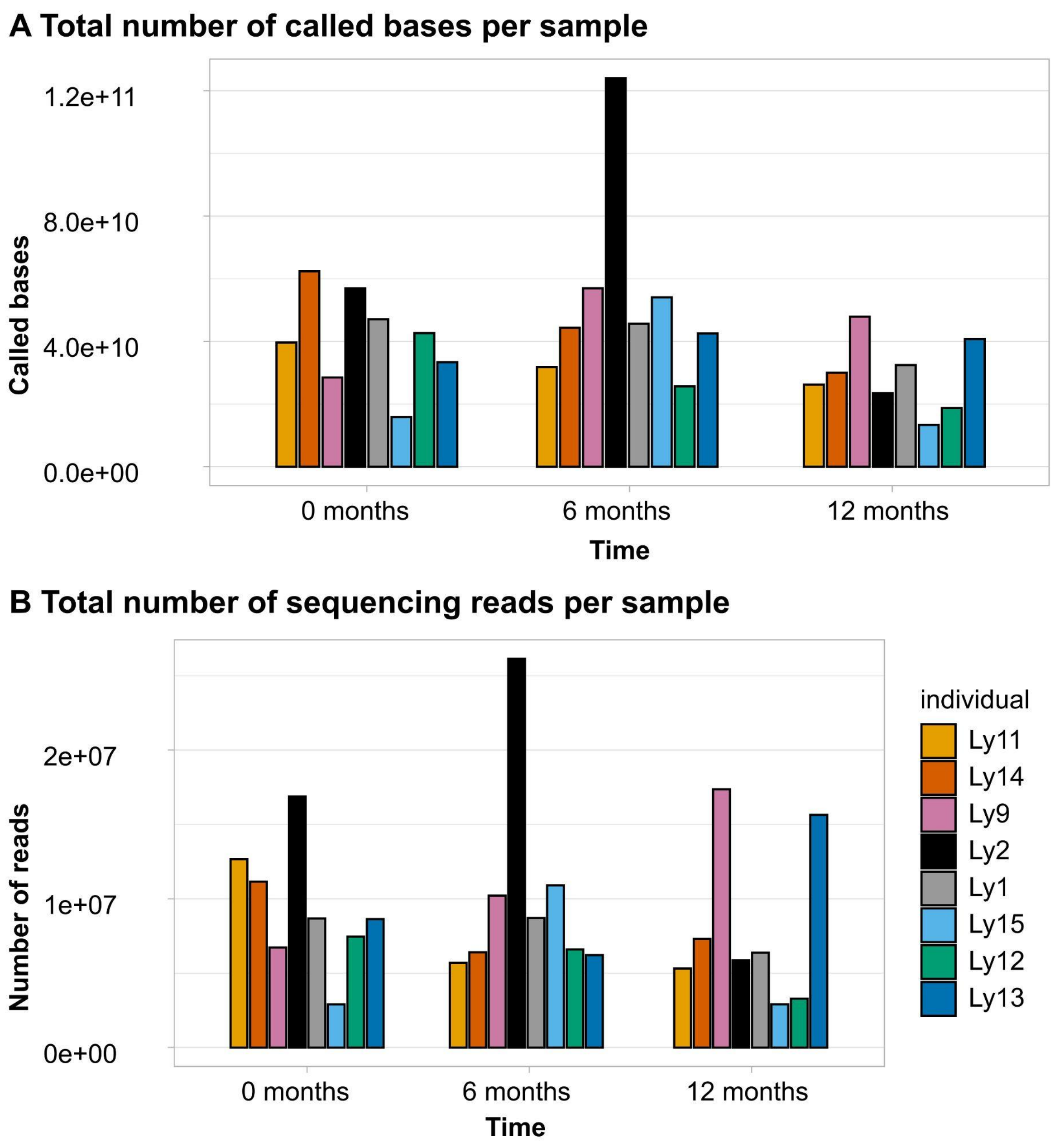

Quality metrics from nanopore Promethion sequencing show the number of called bases per sample **(A)** and the number of reads per sample **(B)** at different time points. Individual Ly15 (light blue) has a lower sequencing quality and differs from other control individuals in main text Figures 2C and 3A.

Supplemental Figure 5. Average change in genomic methylation and hydroxymethylation levels in control and TET2 mutant individuals after 6 months of vitamin C supplementation.

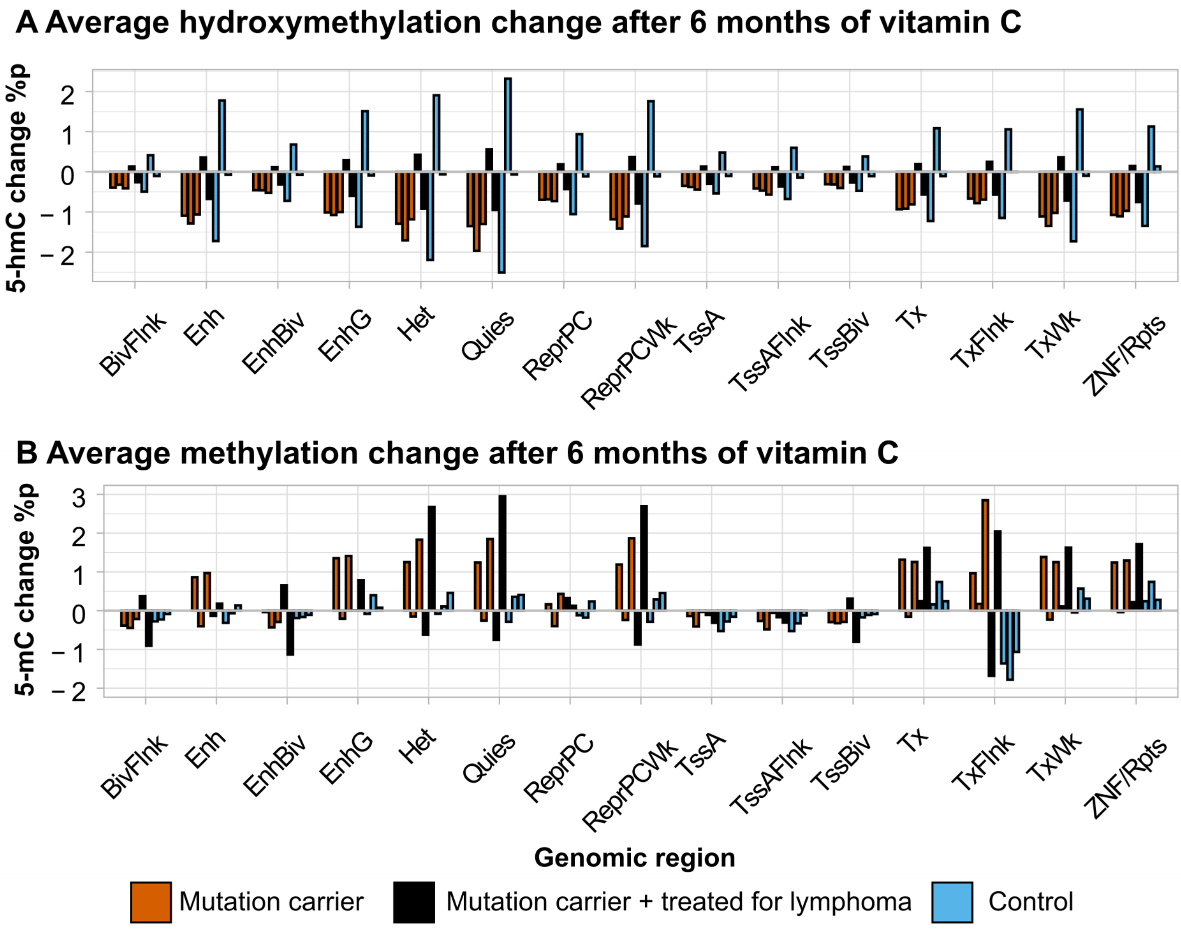

(A) Average change in DNA hydroxymethylation at different genomic regions in PBMCs after 6 months of vitamin C supplementation. **(B)** Average change in DNA methylation after 6 months of vitamin C supplementation. Note the different Y-scales.

Supplemental Figure 6. Proportion of hypermethylated positions at PBMC annotations at different time points.

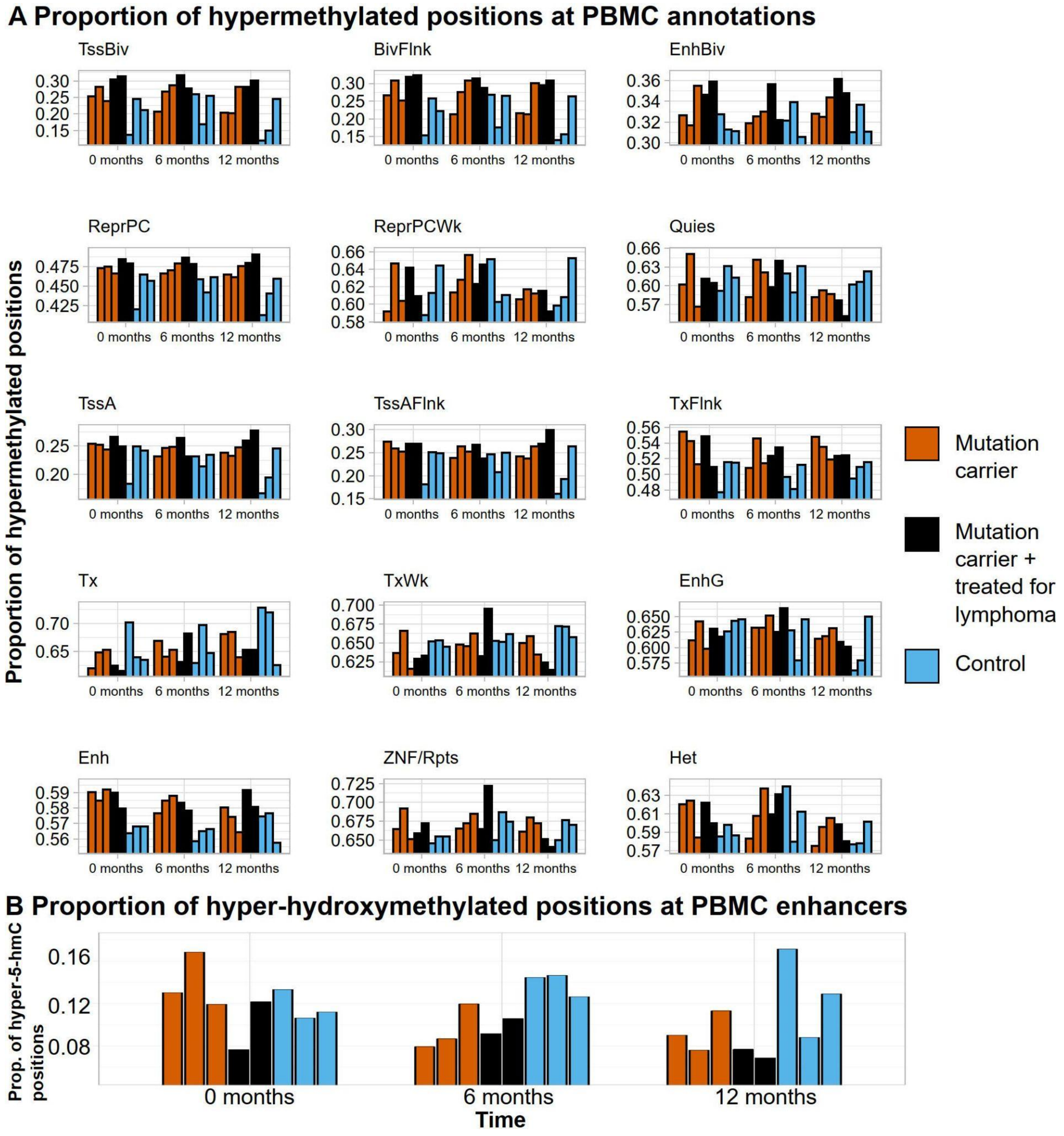

(A) Proportion of hypermethylated positions at PBMC annotations at different time points. Y-axis shows the proportion of hypermethylated positions, x-axis shows the time point. Note the different Y-scales. **(B)** Proportion of loci hydroxymethylated over the sample average at PBMC enhancers at different time points.

Supplemental Figure 7. Average methylation values at different transcription factor binding sites overlapping PBMC enhancers before vitamin C supplementation.

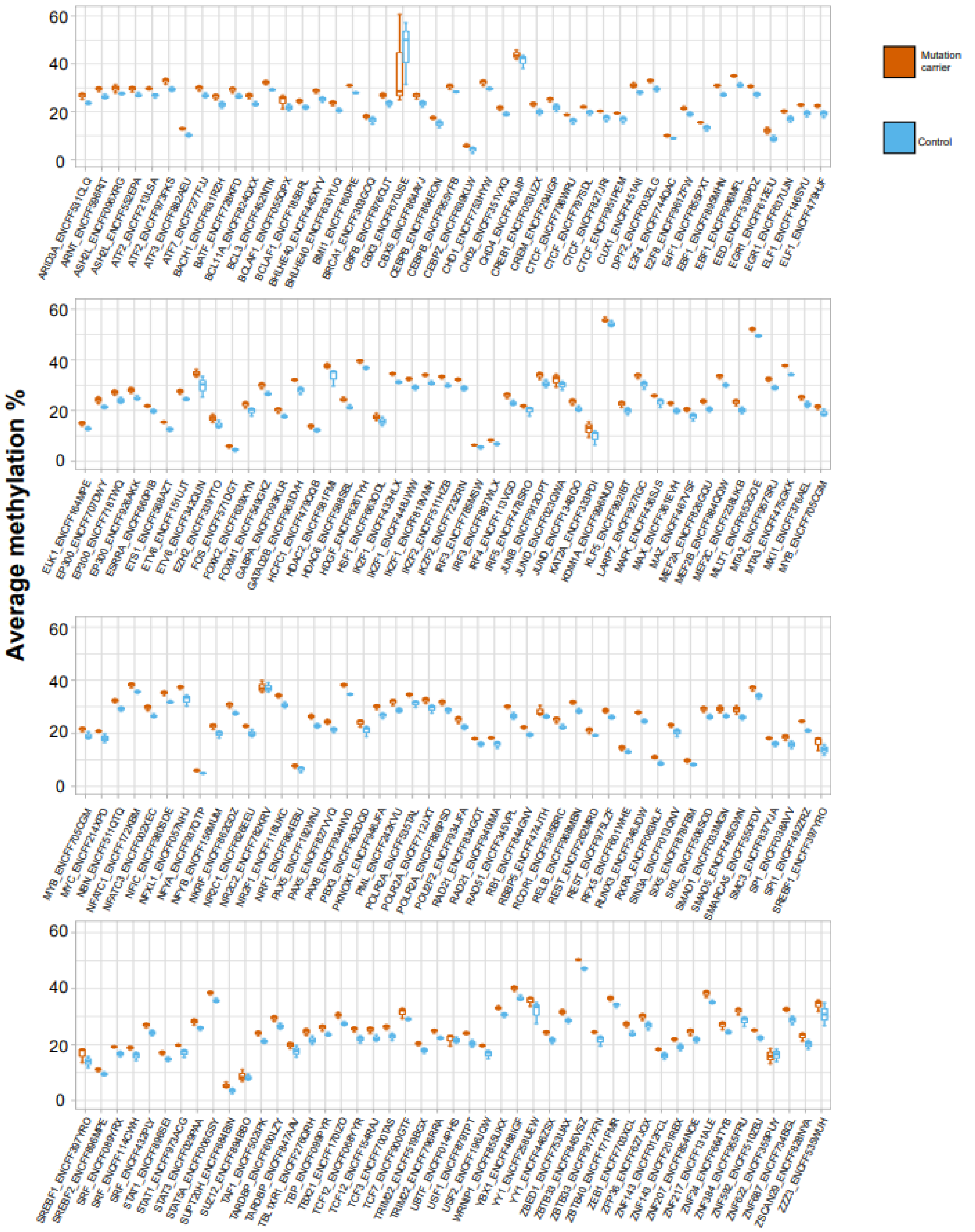

Boxplots showing the average methylation values at different transcription factor binding sites overlapping PBMC enhancers at baseline. TFs are in alphabetical order. Unaffected mutation carriers tend to show higher methylation averages at binding sites of many transcription factors.

Supplemental Figure 8. Effect of *TET2* mutation on methylation at transcription factor binding sites overlapping enhancers.

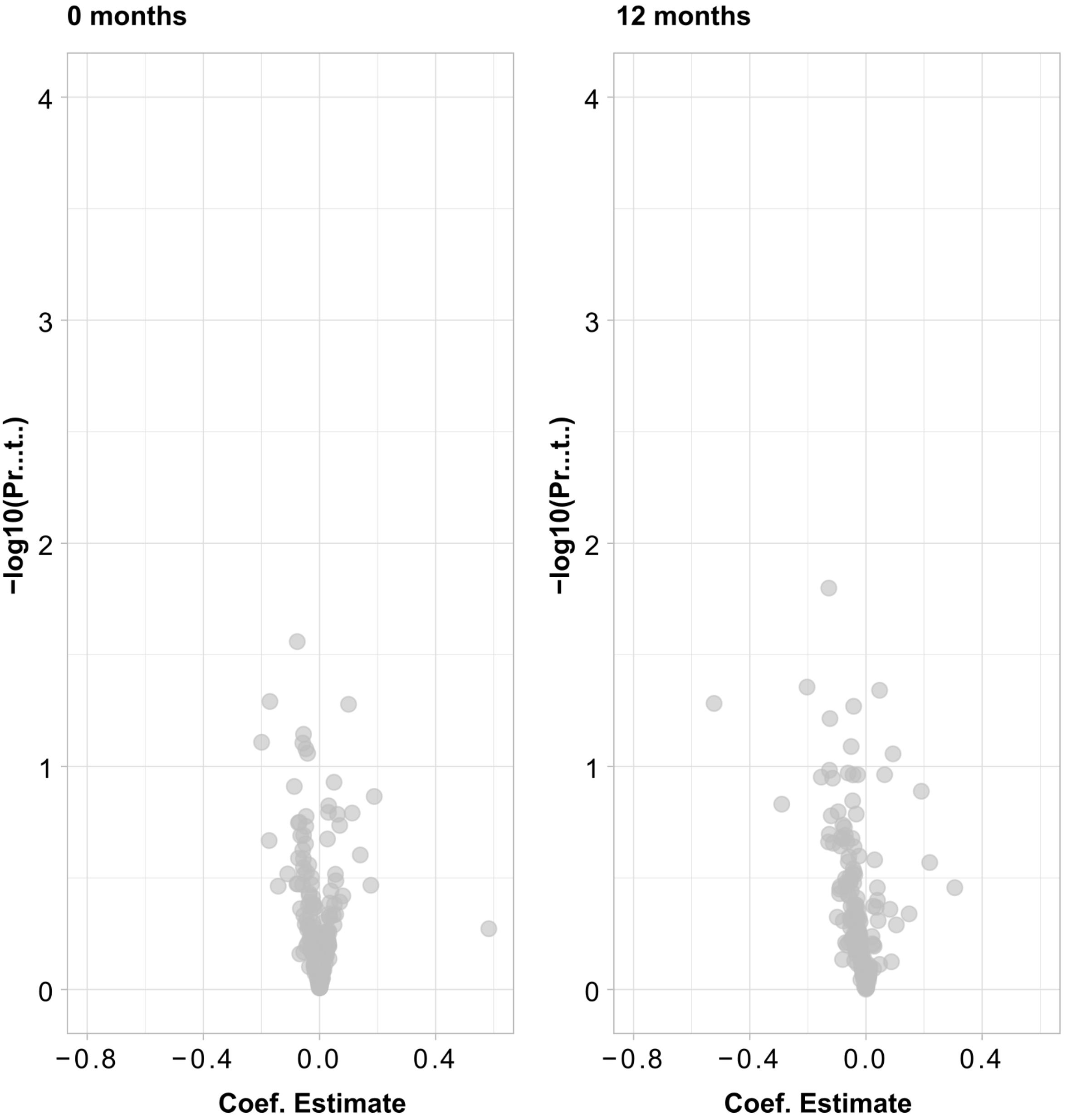

Volcano plots showing the effect of *TET2* mutation status on average methylation at binding sites of different transcription factors overlapping enhancers at **(A)** 0 months and **(B)** at 12 months. In this analysis, the positions were not additionally required to be hypermethylated when calculating the average methylation at transcription factor binding sites. We observed no factors whose methylation level would be significantly affected by the mutation status. Mutations in *TET2* seem to have an effect on methylation levels only at very specific genomic regions (See Main Figure 3A, 5 and earlier studies[7],[8]).

Supplemental Figure 9. Expression of SLC23 ascorbate transporters.

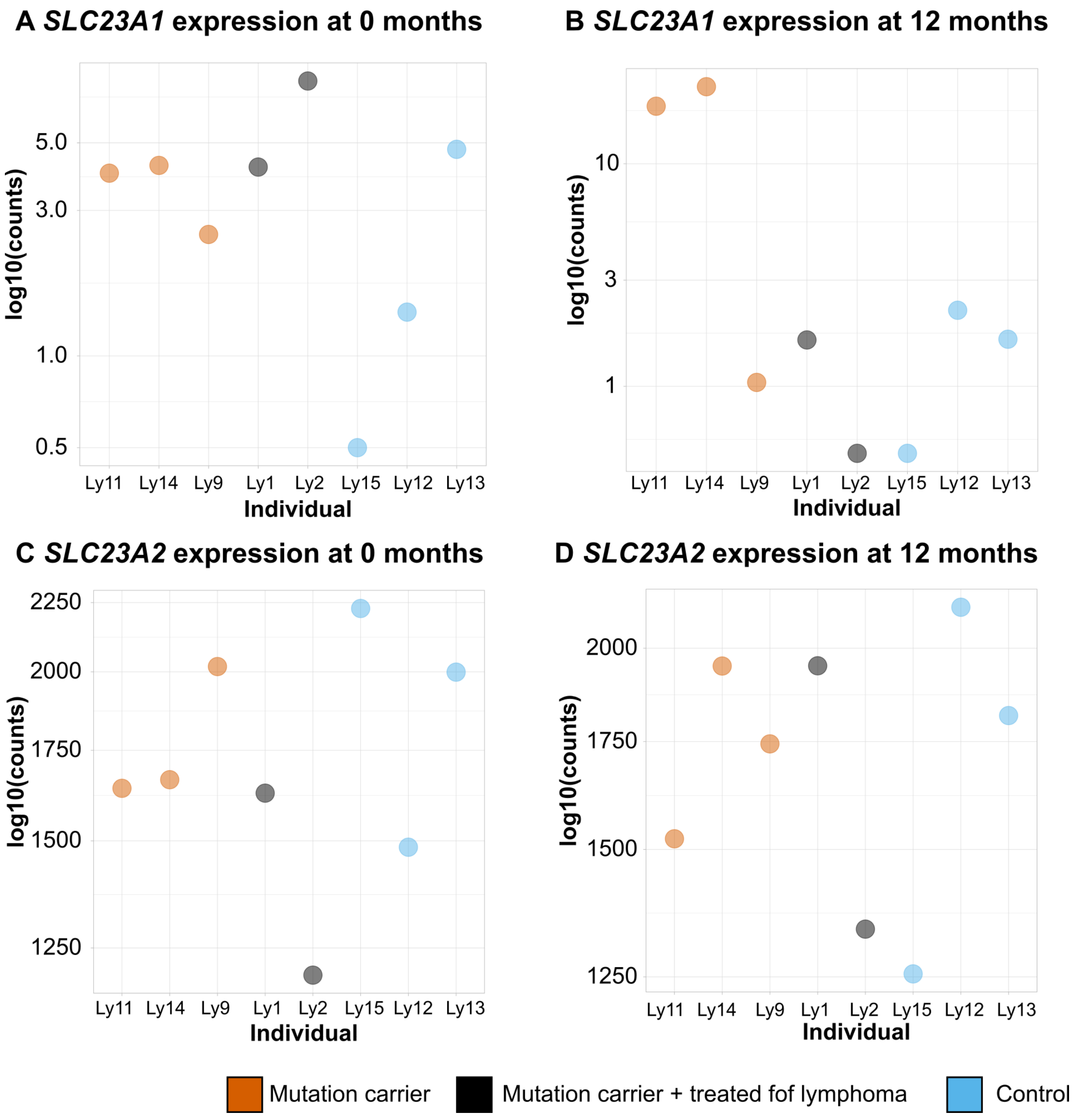

As the transportation of vitamin C to cells is mostly controlled by *SLC23A1* and *SLC23A2[9]* , we visualized the expression of these genes separately. No statistically significant differences were present in the expression of these genes before or after the vitamin C trial. **(A)** *SCL23A1* showed higher expression in the mutation carriers compared to two control individuals, this difference however being nonsignificant. **(B)** The expression of *SLC23A1* seemed to increase after the vitamin C trial in two mutation carriers. Expression of *SLC23A2* showed no clear differences between the mutation carriers and control individuals before **(C)** or after **(D)** the trial.

Supplemental Figure 10. Estimated proportions of cell types in blood from deconvolution of RNA-sequencing data.

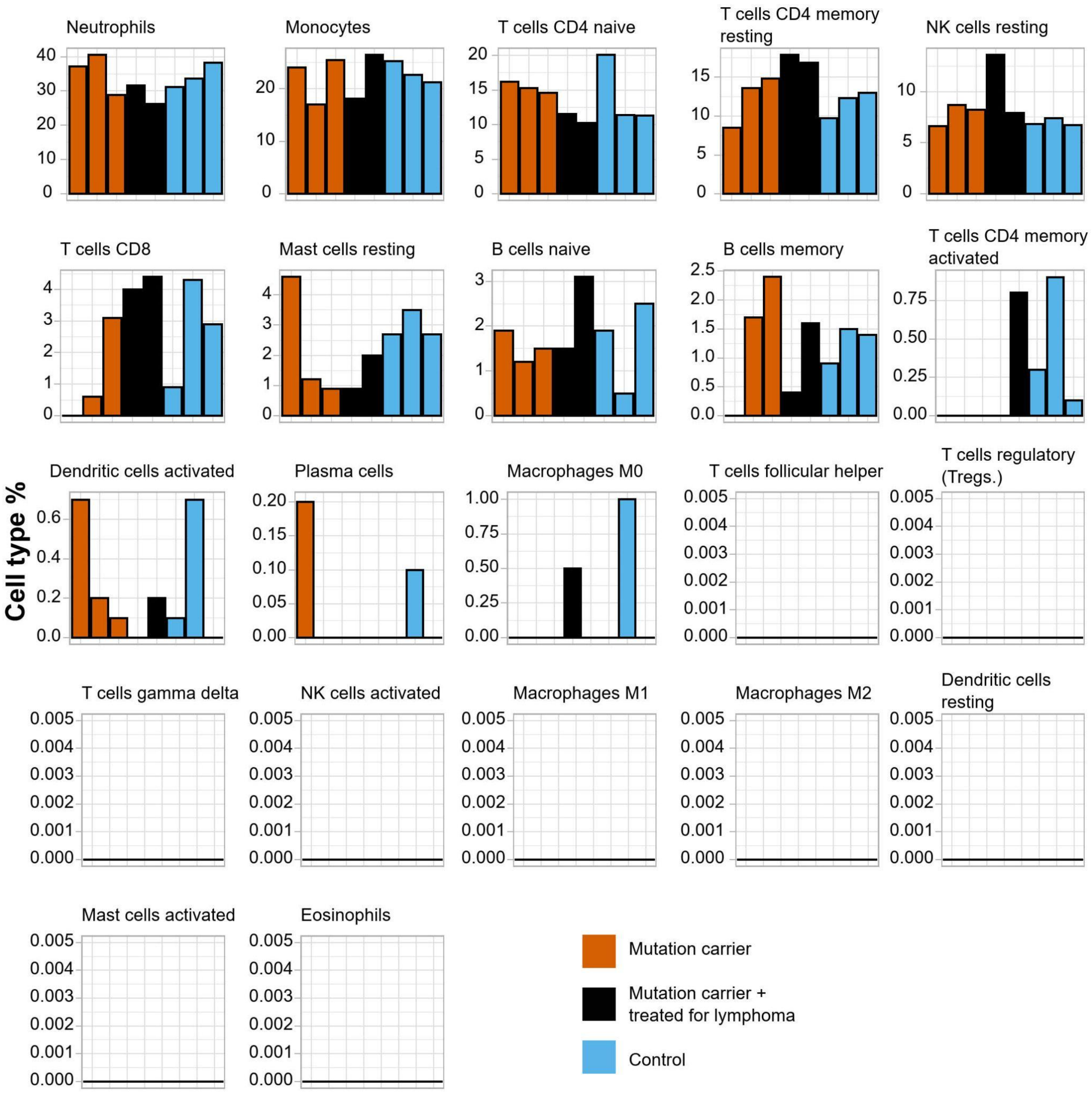

Estimated percentages of 22 different cell types in whole blood samples retrieved before the vitamin C trial based on deconvolution of bulk RNA-sequencing data (see Methods). Neutrophils were the most prevalent cell type in all samples, followed by monocytes and CD4+ T cells. Note the different Y-scales. Cell types with empty plots were not detected in the deconvolution analysis.

Supplementary Figure 11. Changes in estimated cell type proportions after the vitamin C trial.

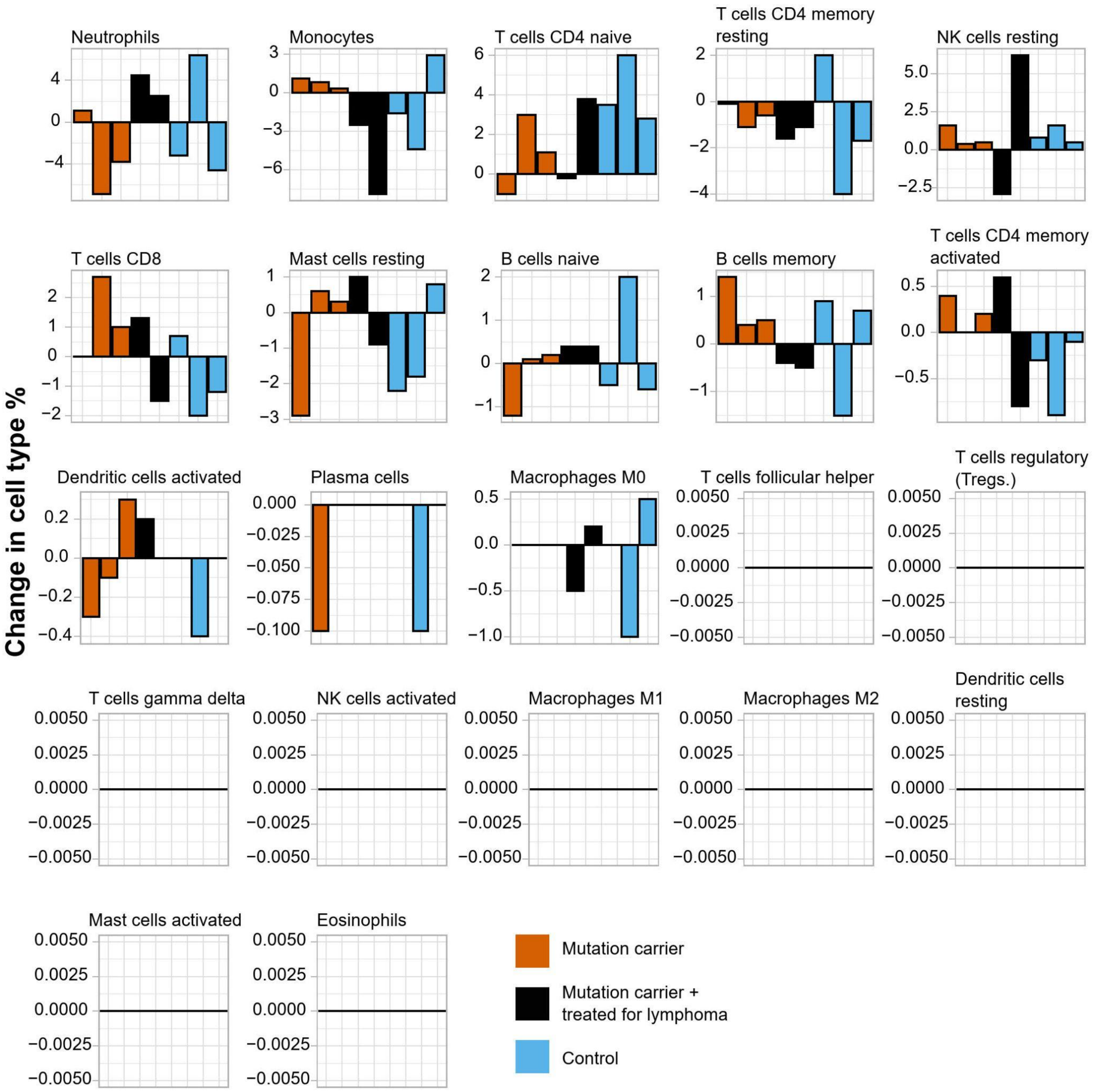

The plots show the changes in the estimated cell type percentage at 12 months compared to estimated percentage at 0 months. A positive number depicts an increase in the cell type proportion while a negative one depicts a decrease. Small changes were present in all cell types which were detected from the bulk RNA-sequencing data at 0 months (Supplementary Figure 10). Changes are not consistent or associated with mutation status, indicating that the observed changes are due to normal variation and not induced by vitamin C.

Supplemental Figure 12. Demethylation model parameter changes in 6 and 12 month timepoints.

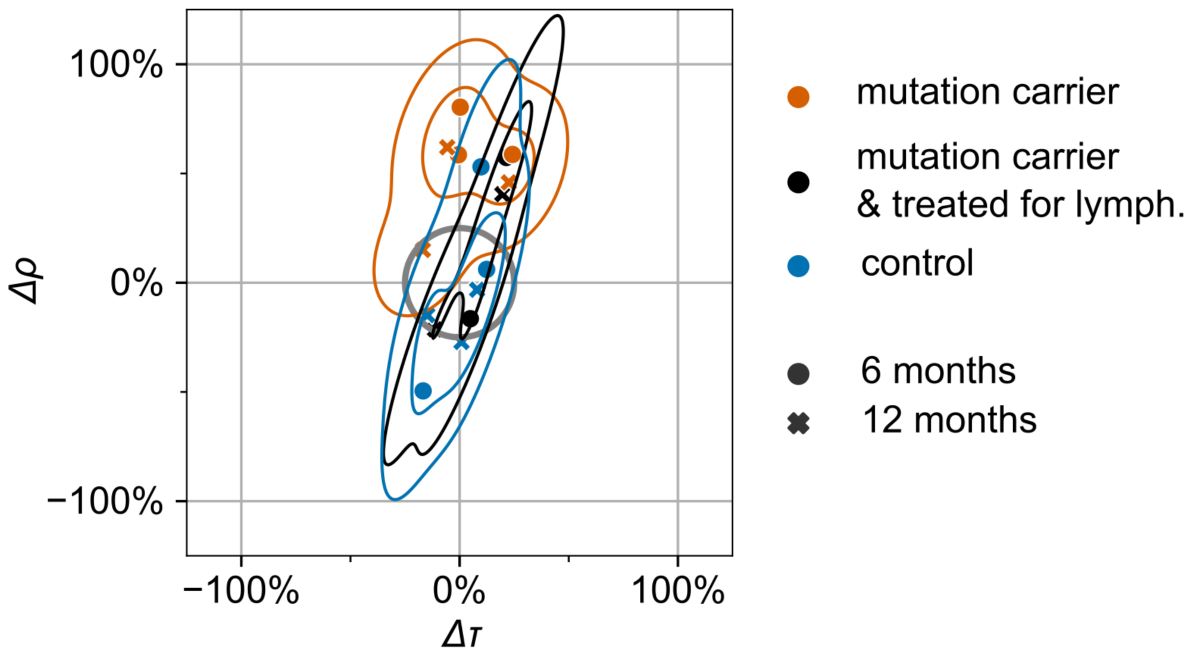

Similar to Figure 6B 2 dimensional plot of the change of rates for the reaction rate model of Figure 6A on PBMC enhancer regions. Each marker stands for an individual, colored by mutation status. The crosses are at percent change of τ (first oxidation step rate) and ρ (further oxidation and repair rate) from 0 to 12 months time point (as arrow heads in Fig. 6B) and circles are respectively for 6 month time points. Contour lines are kernel density estimates for change rates for different groups of individuals, colored the same as the markers. Gray circle is at 25% change as in Figure 6B.
The high density region for rate changes is non-overlapping with zero only on mutation carriers, with Δρ being consistently positive.

References for Supplemental Data

1. Kaasinen E, Kuismin O, Rajamäki K, Ristolainen H, Aavikko M, Kondelin J, et al. Impact of constitutional TET2 haploinsufficiency on molecular and clinical phenotype in humans. Nat Commun. 2019;10:1252.

2. Roadmap Epigenomics Consortium, Kundaje A, Meuleman W, Ernst J, Bilenky M, Yen A, et al. Integrative analysis of 111 reference human epigenomes. Nature. 2015;518:317–30.

3. ENCODE Project Consortium. An integrated encyclopedia of DNA elements in the human genome. Nature. 2012;489:57–74.

4. Davis CA, Hitz BC, Sloan CA, Chan ET, Davidson JM, Gabdank I, et al. The Encyclopedia of DNA elements (ENCODE): data portal update. Nucleic Acids Res. 2018;46:D794–801.

5. Sender R, Milo R. The distribution of cellular turnover in the human body. Nat Med. 2021;27:45–8.

6. Mi H, Ebert D, Muruganujan A, Mills C, Albou L-P, Mushayamaha T, et al. PANTHER version 16: a revised family classification, tree-based classification tool, enhancer regions and extensive API. Nucleic Acids Res. 2021;49:D394–403.

7. Hon GC, Song C-X, Du T, Jin F, Selvaraj S, Lee AY, et al. 5mC oxidation by Tet2 modulates enhancer activity and timing of transcriptome reprogramming during differentiation. Mol Cell. 2014;56:286–97.

8. Rasmussen KD, Jia G, Johansen JV, Pedersen MT, Rapin N, Bagger FO, et al. Loss of TET2 in hematopoietic cells leads to DNA hypermethylation of active enhancers and induction of leukemogenesis. Genes Dev. 2015;29:910–22.

9. May JM. The SLC23 family of ascorbate transporters: ensuring that you get and keep your daily dose of vitamin C. Br J Pharmacol. 2011;164:1793–801.
